# Variability of Biodegradation Rates of Commercial Chemicals in Rivers in Different Regions of Europe

**DOI:** 10.1021/acs.est.4c07410

**Published:** 2024-10-28

**Authors:** Run Tian, Malte Posselt, Kathrin Fenner, Michael S. McLachlan

**Affiliations:** 1 Department of Environmental Science (ACES), Stockholm University, 10691 Stockholm, Sweden; 2 Eawag, Swiss Federal Institute of Aquatic Science and Technology, 8600 Dübendorf, Switzerland; 3 Department of Chemistry, University of Zürich, 8057 Zürich, Switzerland

**Keywords:** biodegradation, OECD 309, spatial variability, organic micropollutant, pristine and contaminated

## Abstract

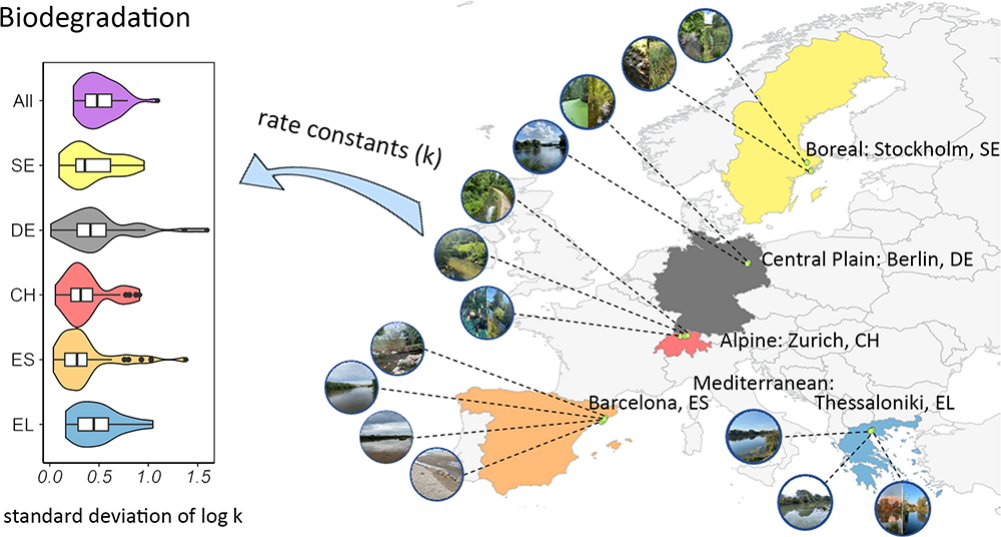

Biodegradation is one of the most important processes influencing the fate of organic contaminants in the environment. Quantitative understanding of the spatial variability in environmental biodegradation is still largely uncharted territory. Here, we conducted modified OECD 309 tests to determine first-order biodegradation rate constants for 97 compounds in 18 freshwater river segments in five European countries: Sweden, Germany, Switzerland, Spain, and Greece. All but two of the compounds showed significant spatial variability in rate constants across European rivers (ANOVA, *P* < 0.05). The median standard deviation of the biodegradation rate constant between rivers was a factor of 3. The spatial variability was similar between pristine and contaminated river segments. The longitude, total organic carbon, and clay content of sediment were the three most significant explanatory variables for the spatial variability (redundancy analysis, *P* < 0.05). Similarities in the spatial pattern of biodegradation rates were observed for some groups of compounds sharing a given functional group. The pronounced spatial variability presents challenges for the use of biodegradation simulation tests to assess chemical persistence. To reflect the variability in the biodegradation rate, the modified OECD 309 test would have to be repeated with water and sediment from multiple sites.

## Abstract 2

The biodegradation rate of organic micropollutants varies between European rivers with a median standard deviation corresponding to a fold difference of 3.

## Introduction

European and North American chemical inventories suggest that there are about 150000 substances in commerce today.^1^ Many find their way into the environment, becoming environmental pollutants. A key question in the regulation of organic pollutants is the reversibility of chemical contamination; if society decides that an environmental contaminant is problematic, then reducing emissions should lead to a reduction in exposure.^2,3^ Biodegradation is one of the most important processes influencing the level and reversibility of contamination,^2^ and the rate of biodegradation is typically one of the largest sources of uncertainty in assessing chemical exposure.^4^ Our initial measurements in two rivers suggest considerable variability in biodegradation rates.^5^ Spatial variability affects how we judge the representativeness of measurements of biodegradation rates and how we extrapolate measurements for larger-scale evaluations of chemical persistence, fate, and exposure. However, a quantitative understanding of the spatial variability of biodegradation rates of diverse chemicals in aquatic systems and the mechanisms that govern this variability is very limited.

Biodegradation results from a complex interplay of several factors, including the bioavailable concentration of the pollutant, bacterial community composition (BCC) and functions, and the mixture of natural and anthropogenic chemicals present.^6^ Different environmental conditions (pH, temperature, dissolved and particulate organic matter, dissolved oxygen, nutrient status, sediment properties, salinity, etc.) influence these factors and hence can affect the biodegradation of chemicals. In addition, human activities (e.g., discharging of contaminants) can modify the BCC in aquatic systems, potentially changing its biodegradation capacity.^7^ Local environmental conditions have been found to impact the species composition of the microbial community,^8−11^ but our understanding of how this influences the ability of the microbial community to degrade contaminants, whether it be through cometabolism or metabolism by specialized organisms, is limited. It follows that it is important to quantify the influence of environmental factors on the biodegradation capacity of rivers. To date, only a few studies have investigated the spatial variability in biodegradation rates of chemicals and the influence of environmental factors,^12−15^ and none have covered a large and diverse set of chemicals and an extensive spatial range.

The present study is an attempt to begin addressing this knowledge gap. We decided to focus on freshwater systems because in the European chemicals legislation REACH biodegradation in freshwater is prioritized in assessing chemical persistence,^16,17^ and we further constrained ourselves to rivers. We chose to study spatial variability at the regional scale, covering a wide range of climatic and geographical conditions such as temperature, solar irradiation, and sources of organic matter that are likely to shape riverine ecosystems. Our aim was to establish the range of biodegradation that can be expected across Europe. We determined primary biodegradation rates, as this allowed us to study multiple chemicals simultaneously, and we are particularly interested in how consistent the spatial variability in biodegradation rate is within and across chemical groups. Our main objectives were to (i) quantify the spatial variability in biodegradation rates in rivers for multiple chemicals, and (ii) identify the environmental factors that best explain the spatial variability in biodegradation rates. We conducted a series of modified OECD 309 experiments that were designed to maximally simulate field conditions. The data set used to assess the spatial variability consisted of the first-order rate constant for primary biodegradation of 97 chemicals in 19 river segments located in five countries, including the Mediterranean (Spain and Greece), alpine (Switzerland), central plain (Germany), and boreal (Sweden) geographic regions. In addition, we explored the biodegradation behavior of both pristine and contaminated river segments in each country to investigate the impact of anthropogenic pollution on the spatial variability of the biodegradation of chemicals.

## Materials and Methods

### Selection and Sampling of European River Segments

Due to the limited information on what environmental variables are important determinants of biodegradation rates in rivers, we conducted a range-finding study including rivers from regions with different climate and geographical characteristics, taking into consideration accessibility to sampling sites and the possibilities for rapid transport of samples back to the laboratory. Nineteen river segments were sampled, located in four European regions (five countries, Figure S1): Mediterranean (four in Spain, ES and four in Greece, EL), alpine (four in Switzerland, CH), central plain (three in Germany, DE), and boreal (four in Sweden, SE). Their latitude and longitude ranged from N40° to N60° and E1° to E23°, covering a broad range of climatic and geographic characteristics in Europe (Table S1).

In each country except ES, we obtained at least one pair of river segments from upstream and downstream of a wastewater treatment plant (WWTP). All upstream river segments were upstream of the first WWTP on the river and hence were expected to have received minimal wastewater input. They are referred to as pristine river segments. In ES, we sampled a pristine estuary (ES_F_Pris_Salt). All contaminated river segments except EL_A_Contam were located 0.5–10 km downstream of WWTPs. EL_A_Contam received large volumes of treated and untreated wastewater from large cities, while EL_L_Contam was more strongly impacted by industrial effluents.^18^ For a detailed description of the sampled river segments, see Section S1 of the Supporting Information (SI).

Temperature had been reported to be an important variable affecting biodegradation rates,^19,20^ but we investigated this in a parallel study.^5^ To facilitate the exploration of other elements of spatial variability, we therefore timed the sampling so that the temperature was similar at all sites. Since we were working in different climate zones, this resulted in the sampling stretching from high summer to midautumn of 2022 (from July to October).

Water parameters (temperature (*T*), pH, dissolved oxygen (DO), and electrical conductivity (EC)) were measured on-site using calibrated hand-held probes (HQ portable meters, HACH). The sampling followed the protocol of our previous study.^21^ Briefly, samples of surface water and the top 3–5 cm of sediment were collected and transported in insulated containers from the sampling sites to the lab. The changes in the temperature of the samples during transport were <1 °C. Sampling in each country was completed within 6 h, and the incubations were started within 24 h of sampling.

### Test Compounds

For the biodegradation experiments, an aqueous mixture of 129 test compounds was prepared. The test compounds were the same as in our previous study and showed a broad range of biodegradability.^5^ About 80% of the test compounds have a log *D*_*OW*_ < 3 at pH 7.4 and are found in municipal wastewater and surface water. They comprise multiple use classes (pharmaceuticals, agrochemicals, cosmetics, food additives, and industrial chemicals). Additionally, they were chosen to cover a broad range of molecular structures (sulfonamides, thioethers, phenylureas, amines, etc.). Lists of the test compounds and labeled internal standards used to evaluate the stability of instrumental analysis between samples are provided in Tables S3 and S4.

### Biodegradation Experiments

The experimental setup followed the protocol of an OECD 309 test with slight modifications,^5,21^ which we have previously argued provides better environmental relevance than the standard OECD 309 test.^21^ Briefly, sediment was sieved to 2 mm and homogenized, added to river water (50 g wet solid L^–1^), and kept in suspension with an orbital shaker. Test treatments (TEST, three replicates) and abiotic controls (two replicates with sediment (sorption controls, SC) and one sediment-free hydrolysis control) were spiked with an aqueous solution of 129 compounds to a concentration of 1 μg L^–1^ each. The SCs were for distinguishing biodegradation from sorption and abiotic degradation, while the hydrolysis control was for distinguishing sorption from abiotic degradation. All abiotic controls were sterilized by adding 0.1% sodium azide. Dissipation of the test compounds was monitored by analyzing filtered (0.45 μm PTFE filters) subsamples taken from the water phase of each vessel (1.5 mL in duplicate) after 0, 2, 5, 9, 18 h, 1, 2, 4, 7, and 10 days. The spiked incubations were shaken for 10 min before the first sample was collected to ensure homogeneous distribution. All experiments were carried out in the dark at river water temperature (20.5 ± 3.5 °C). More details on the experimental setup can be found in section S3 of the SI.

During incubation, aqueous pH, temperature, and EC were measured manually on each sampling day in each incubator, and DO was measured on a daily basis. The particle size distribution (including the content of clay, silt, and sand) of sieved and homogenized sediment was measured using a Mastersizer 3000 (Malvern Instruments Ltd., Malvern, UK). Total organic carbon (TOC) was measured in sieved and homogenized sediment by Eurofins Environment Testing Sweden AB using the loss-on-ignition method.^22^ Cell density measurements were conducted for the sieved and homogenized sediment and river water prior to filling the incubator using a BD Accuri C6 Flow Cytometer (BD, Belgium). Sampling and sample preparation for cell density analysis were based on our previous study.^5^ The results were used to calculate total cell counts (TCC) in the incubator. The TOC, TCC, and particle size distribution data can be found in Table S2.

### Chemical Analysis and Data Processing

Chemical analysis followed the procedure described in Tian et al.^21^ using an ultrahigh-performance liquid chromatography system coupled to a Q Exactive HF Hybrid Quadrupole-Orbitrap mass spectrometer (UHPLC-Orbitrap-MS/MS, Thermo Fisher Scientific, San Jose, CA) with electrospray ionization (ESI). Data processing was carried out in Compound Discoverer 3.3. All data were drift-corrected based on a quality control sample that was measured between every six samples. The drift correction was conducted using the R package *batchCorr*.^23^ A 15-point matrix-matched calibration curve was measured with concentrations ranging from 1 ng L^–1^ to 10 μg L^–1^. The limit of detection (LOD) was calculated based on the lowest detected calibration standard. The lowest detected calibration standard within the linear range was set as the limit of quantification (LOQ). The rate constant was classified as “not available” when no data were found above the LOQ. The estimation of the observed degradation rate constant (*k*_observed_) was performed by a Python algorithm (Chowclassifier) as described by Tian et al.^5^ The evaluation of the kinetics was based on the peak area as the variability in response factor between samples was low for most analytes (e.g., the median relative standard deviation (RSD) of the peak area of the internal standards between the samples from the test treatments was 9%). Before calculating *k*_observed_, the data from the test treatments were corrected for dissipation in the SC as described in our previous work.^5^ The value of *k*_observed_ was calculated using linear least-squares regression of the natural logarithm of the chemical’s peak area versus time from the start of the incubation as long as dissipation was first order (Figure S3, Section S4 of SI). All data from the three replicate incubations were used simultaneously in the Python algorithm, and only data above the LOQ were included. When biphasic kinetics were observed, the initial rate constant was used. The estimated initial *k*_observed_ was considered valid when it was based on at least five data points and significantly different from zero (*P* < 0.05). About 1.7% of the rate constants were based on just five data points, but none of those were outliers (>1.5 times the interquartile range) with respect to the valid rate constants for the other river segments. When no valid *k*_observed_ could be calculated for a given compound (i.e., when one of these two conditions was not satisfied), we used a gap-filling approach employing the following decision chain to ensure that the gap-filled *k*_observed_ were consistent with the observations (a flowchart of the gap-filling process is shown in Figure S4):

1. if the 99% confidence interval of estimated *k*_observed_ (invalid) contains the median valid *k*_observed_ (for the same compound, all other river segments), replace with median valid *k*_observed_ (same compound, all other river segments), otherwise
2. if the 99% confidence interval of estimated *k*_observed_ (invalid) contains the minimum valid *k*_observed_ (for the same compound, all other river segments), replace with minimum valid *k*_observed_ (same compound, all other river segments), otherwise
3. gap fill with the minimum valid *k*_observed_ (all compounds, all river segments).

For chemicals that sorb, the bioavailable fraction in the dissolved phase is reduced. Consequently, the observed rate of decrease in the dissolved concentration is slower than it would be if there was no sorbed chemical. Therefore, *k*_observed_ was corrected to yield *k*, the rate constant that would hypothetically be observed if all of the chemical was fully dissolved:^24^
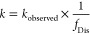
1where *f*_Dis_, the fraction of compound in the dissolved form, was estimated as the quotient of the peak area of the test chemical between the SC and the hydrolysis control at *t* = 0 (i.e., 10 min after adding the standard mixture). This procedure was possible because, based on the peak areas of the internal standards, the matrix effects were similar in these two controls.

Given the evidence indicating that, in the case of ionizing substances, changes in the neutral fraction (*f*_N_) change the fraction of chemicals available for cellular uptake,^24−27^*k* was further corrected to a reference pH of 7 to allow better comparison of the biodegradation efficiency of the microbial communities across sites with varying pH:
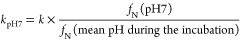
2*k* and *k*_pH7_ were used to explore the spatial variability in biodegradation rates between the river segments and their association with different environmental factors.

The values of *k*_observed_, *k*, *k*_pH7_, *f*_Dis_, and *f*_N_ are given in the Supplemental Data Set S1. Dissipation in SC and the consequences for the estimation of *k*_observed_ are detailed in Section S6 of the SI. The results for the Swedish river segments were obtained from our previous study.^5^

### Statistics and Reproducibility

In order to assess the reproducibility of the biodegradation test and evaluate the statistical significance of differences in biodegradation rate constants using analysis of variance (ANOVA), we also calculated *k* for each individual replicate incubation using linear regression. Principal component analysis and redundancy analysis were performed using the Vegan package (v.2.6.4)^28^ in R (v.4.3.1). Hierarchical clustering was performed using the R package ComplexHeatmap (v.2.16.0).^29,30^ Spatial variability was assessed using the standard deviation (stddev) of the logarithm of the rate constant (the antilog of this number gives the standard deviation as a fold difference). Stddev (log *k*) was used to describe the spatial variability in biodegradation rate as observed in the environment, while stddev (log *k*_pH7_) was used to explore different explanations for this variability as it was already corrected for one important cause of variability (*f*_N_).

## Results and Discussion

### Quality Assurance of European Data set

The measured environmental factors (pH, DO, EC, TOC, particle size distribution, and TCC in water and sediment) varied widely across the 19 river segments (Section S1 of SI). Overall, the conditions in the incubation flasks mirrored the environmental conditions well (except for DO which always increased to saturation before the experiments), and they were similar between the three TEST replicates (Figure S6 of SI).

Out of 129 spiked compounds, 97 were quantified, and 95 were biodegraded in at least one river segment, while two (C_12_ isethionate, and hydrochlorothiazide) had rate constants that were not significantly different from zero in all river segments during the 10-day incubation (Supplemental Data Set S1). Compound dissipation in SC was at least 2.5 times slower compared to dissipation in TEST for 90% of the *k*_observed_ estimations (Section S6 of SI). Only 13 compounds exhibited significant sorption to the sediment (*f*_Dis_ < 0.5) in at least 25% of the river segments (Supplemental Data Set S1). All of them are bases and the same phenomenon was observed in our previous study.^5^ The RSD of *k*_observed_ between the 3 replicates was <30% for 80% of the degraded chemicals. The level of precision agreed with our previous evaluation of the modified OECD 309 test.^21^ The pH varied by 1.6 pH units between the rivers (Table S2 of SI). Of the 97 quantified compounds, 92 are ionizable, and the *f*_N_ of 55% of them varied markedly (a factor of 2 or more) across the observed pH range. *k* was corrected to account for the effect of changing speciation by normalizing *k* to pH7 (eq 2).

One of the studied river segments was an estuary in Spain (ES_F_Pris_Salt). Half of the quantified compounds did not have a valid *k* at this site. This site was clearly different from the freshwater sites with an EC (salinity) 30 to 100 times higher (Section S1 of SI), and it was excluded from the evaluation of the spatial variability.

### Are Biodegradation Rate Constants Similar in Different European Rivers?

The values of *k* varied by almost 5 orders of magnitude, from 0.001 to 68.6 d^–1^. For a given compound, valid *k* values varied by up to 3 orders of magnitude between river segments within a certain country and by up to 4 orders of magnitude across all river segments (Supplemental Data Set S1). Ninety-two out of 97 compounds had valid *k* in at least two river segments, and all of them, except for IRE, showed significant differences in valid *k* and *k*_pH7_ across the 18 freshwater segments (ANOVA, *P* < 0.05, Figure S7). Small variability in the dissipation rate constant of IRE was found in a previous study using the OECD 308 test.^31^ The standard deviations of log *k* and log *k*_pH7_ across the European river segments were similar, with a median value for all compounds of 0.46 (a factor of 3). This agrees with our previous observation on the seasonality of biodegradation rates,^5^ which suggested that the spatiotemporal variability of biodegradation rates was not reduced by correcting for differences in the neutral fraction. As there were only 11 compounds (CAF, CIL, IPO, KET, LIN, MEC, MPL, RAN, RUF, SOT, TAE) that had valid *k* in all 18 river segments, gap-filling was performed for 86 compounds for at least one river segment. After gap-filling, the standard deviations of log *k* and log *k*_pH7_ increased to about 0.61 (a factor of 4). Gap-filling *k* increases the uncertainty in the spatial variability, while neglecting river segments with nonsignificant *k* leads to an underestimation of the spatial variability in the biodegradation rates. Therefore, we chose a compromise for our further evaluation of spatial variability. We used only the compounds that were gap-filled for less than one-third of the river segments (65 out of 97 compounds).

Using this data set, we calculated the stddevs of log *k* and log *k*_pH7_ between river segments within each country and across all countries. The stddevs of log *k* and log *k*_pH7_ were similar for a given compound (the median of the absolute difference between the paired values was 0.006), and each had a median value of about 0.5 (a factor of 3) across the 65 compounds. For the rest of the paper, we work with *k*_pH7_ to explore different explanations for the spatial variability in the biodegradation rate. The results of *k* are provided in Section S8 of the SI.

**Figure 1:**
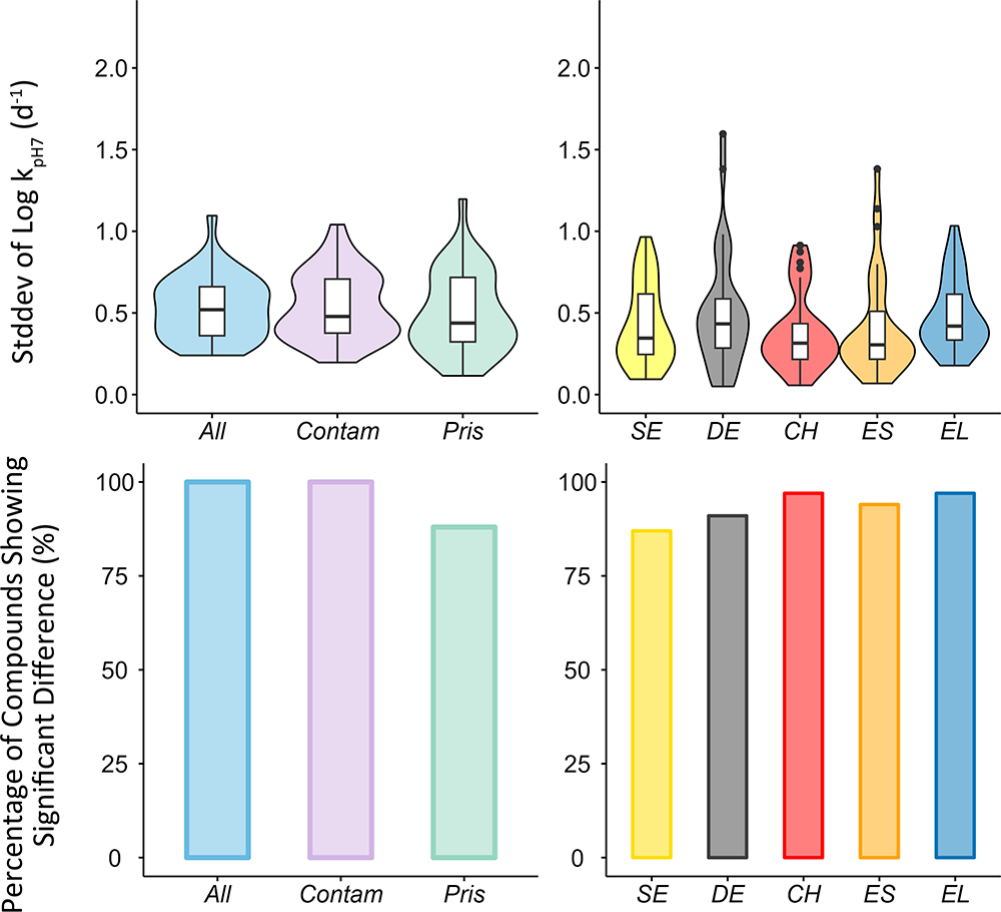
Violin plots show the median, 25th, and 75th percentiles of the standard deviation (stddev) of log *k*_pH7_ (d^–1^). Histograms show the percentage of compounds that displayed significant differences (ANOVA, *P* < 0.05) across the river segments. The results are presented for all 18 river segments (All), as well as the following subsets: 13 contaminated river segments (Contam), and five pristine river segments (Pris), in five European countries (SE, DE, CH, ES, EL). Only compounds that had valid *k*_pH7_ in at least 12 out of 18 river segments and had *k*_pH7_ in all 18 river segments after gap-filling were used for comparison. The points that lie above the whisker are outliers.

The stddevs of log *k*_pH7_ across the 18 river segments varied from 0.24 to 1.1 (a factor of 1.7 to 12, Figure 1), suggesting that the spatial variability was small for some compounds, while large for others. The median stddevs of log *k*_pH7_ within each country varied from 0.30 (ES) to 0.42 (EL), and 87% (SE) to 97% (EL) of the compounds showed a significant difference between river segments within a given country (ANOVA, *P* < 0.05). The results suggest that both regional and local differences contributed to the spatial variability. The spatial variability was similar between pristine and contaminated river segments (Figure 1). Four compounds (ATE, BIS, DIU, and FUR) that showed significant differences between contaminated river segments showed no significant differences between pristine river segments, which indicates that the WWTPs introduce variability in the biodegradation rate constant for some compounds.

### What Environmental Factors Are Associated with the Spatial Variability in Biodegradation Rate Constants?

In order to evaluate the influence of different environmental factors on the spatial variability in biodegradation rate constants among 18 European river segments, a redundancy analysis (RDA) was performed. Of the 11 environmental factors that were available (longitude, latitude, TCC, TOC, EC, DO, pH, 1/*T*, and sand, silt, and clay content), 10 variables were used for RDA analysis. Silt content was excluded as it was significantly correlated with clay and sand content (Pearson’s, *P* < 0.001). Permutational multivariate analysis of variance (999 permutations) was used for significance tests for the RDA model. Envfit (a function that fits environmental vectors or factors onto an ordination) was used to perform significance tests for each explanatory variable (999 permutations).^28^ The percentage of the variance explained (*R*^2^) was adjusted to measure the unbiased amount of explained variance. The statistical test does not accept data gaps. Therefore, to include information for more compounds, we further gap-filled the data set used in the last section, this time focusing on the “not available” data which were replaced with the median *k*_pH7_ within a given country when the stddev of log *k*_pH7_ within the country was <0.4. When adding compounds in this manner, we employed the same inclusion criterion, namely valid data for at least 12 of 18 river segments (i.e., gap-filling for less than one-third of all river segments).

**Figure 2:**
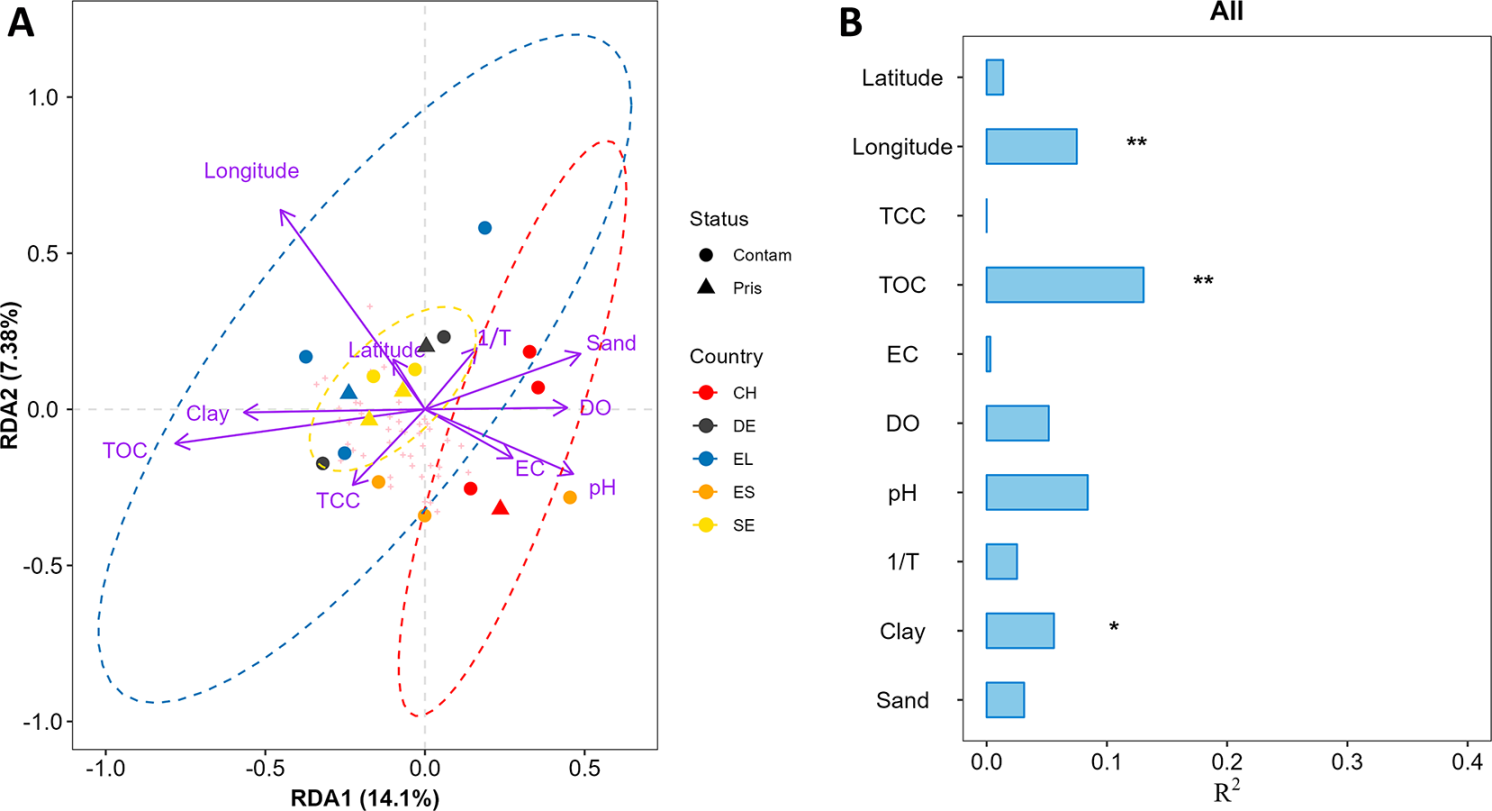
Redundancy analysis (RDA) shows the relationship between log *k*_pH7_ (d^–1^) and 10 environmental factors in 18 river segments in five European countries (SE, DE, CH, ES, EL). The scatterplot (A) shows the first (RDA1) vs the second (RDA2) dimension. The bar chart (B) shows the percentage of the variance explained (*R*^2^, unbiased, Envfit) for each environmental factor. Only compounds that had valid *k* in at least 12 river segments were used for the RDA. The significance of the variance explained was represented by “**” → *P* < 0.01, and “*” → 0.01 ≤ *P* < 0.05 (Envfit). We included 10 environmental factors for RDA: total organic carbon (TOC), electrical conductivity (EC), dissolved oxygen (DO), pH, temperature (1/*T*), particle size distribution (clay and sand content) of sediment, and total cell count in the incubator (TCC). The values of all environmental factors except pH and temperature were log-transformed. Temperature was transformed to 1/*T* (K) to create a distance scale consistent with the Arrhenius relationship.

The results of the RDA analysis for log *k*_pH7_ are shown in Figure 2. An analogous assessment using log *k* yielded similar results (Figure S9). The model significantly explained 41% (unbiased) of the variance in log *k*_pH7_ (*P* < 0.01), while 59% remained unexplained. The first two constrained axes together explained 21%, with RDA1 explaining 14% (Figure 2A). The length of the arrow in the RDA plot indicates the degree of correlation between different explanatory variables and the biodegradation rate, with adjusted *R*^2^ ranging from 0.003 to 0.13. The clustering of countries was weak (Figure 2A); the biodegradation behavior of river segments in different countries overlapped with each other, with clear separation only between CH and SE/DE/EL. The sand content, DO, pH, clay content, and TOC were the environmental variables contributing most to the separation (Figure 2A). Since the pH values in most of the river segments in EL and CH were similar (Table S2), their separation was not a result of bias caused by pH correction. Compared to rivers in other countries, the Swiss rivers studied all have gravel beds which made them more turbulent. This could increase the hyporheic exchange, and make the biodegradation behavior of Swiss rivers distinctive.

The RDA analysis indicated that TOC, longitude, and clay content significantly contributed to explaining the spatial variability of *k*_pH7_ among all 18 river segments (*P* < 0.05, Figure 2B), with TOC being the most important factor (Figure 2B). TOC has been found to influence microbial community dynamics and associated biodegradation in rivers.^32,33^ Particle size distribution of sediment was correlated with log *k*_pH7_ and it has also been found to influence sediment microbial communities and the rates of microbial ecosystem processes.^34^ We did not find a significant correlation between 1/*T* (K) and log *k*_pH7_, which indicates that the variation in biodegradation did not follow an Arrhenius relationship with temperature. This agrees with our previous observations when investigating the seasonality of biodegradation rates.^5^ Notably, we did not observe a significant correlation between TCC and log *k*_pH7_. We have previously observed that TCC was not a major factor explaining the seasonality of biodegradation rates.^5^ The absence of a correlation between spatiotemporal variation in biodegradation rates and TCC might point toward specific enzymes or microorganisms that were responsible for the biodegradation in different river segments. However, the results could also be a consequence of uncertainties in the TCC measurements caused by incomplete and inconsistent detachment of bacteria from sediment during pretreatment. Interestingly, we did not observe latitudinal gradients of biodegradation rates. The regional differences in biodegradation that we observed may reflect the biogeography of BCC, but the decisive features of BCC do not appear to be significantly influenced by latitude on the geographical scale tested in our study. Although the exact nature of the latitudinal gradient in BCC is still somewhat uncertain,^9,10^ it was found to be weak in some global studies.^8,11^ However, the correlation between BCC and biodegradation rates still needs further exploration.

### Do Different Compounds Show Similar Spatial Variability?

**Figure 3:**
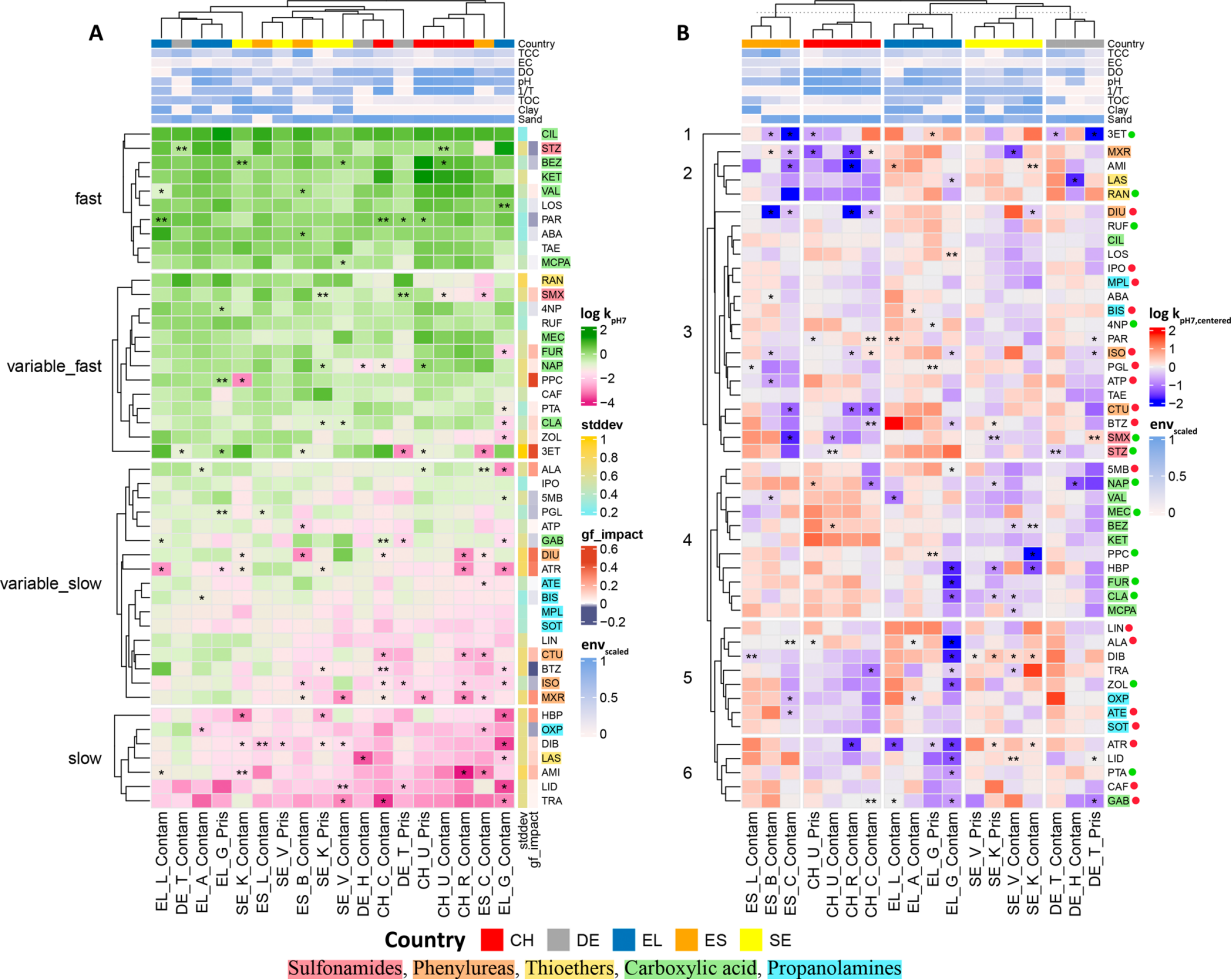
Clustered heatmaps showing A: log *k*_pH7_ (d^–1^) and B: centered log *k*_pH7_ (d^–1^). Only compounds that had valid *k* in at least 12 river segments were studied. Invalid *k*_pH7_ was gap-filled and marked by *, and unavailable *k*_pH7_ was gap-filled and marked by **. In panel A, both the chemical and river segment axes are clustered according to the magnitude of log *k*_pH7_. Annotations on the right depict the spatial variability (stddev of log *k*_pH7_ between river segments) and how it was impacted by gap filling (gf_impact, the difference in the stddev of log *k*_pH7_ before and after gap-filling). In panel B, the chemical axis is clustered according to centered log *k*_pH7_ and the river segment axis is manually grouped according to country. Annotation on the top of both panels depicts the level-scaled values of environmental factors except for longitude and latitude (on a log scale in all cases). Compounds sharing specific functional groups were assigned a color, and this color was used to highlight the chemical name abbreviations. Compounds belonging to the “variable_fast” and “variable_slow” clusters in plot A were labeled in plot B with green and red dots next to the compound name, respectively. For the elaboration of the country and river segment name abbreviations see Table S1. For elaboration of the compound name abbreviations see Table S3.

To better understand the spatial variability in biodegradation rates at an individual compound level, we employed hierarchical clustering using the same data set as used in the last section. The compounds were first grouped according to the magnitude of log *k*_pH7_ (Figure 3A). The median *k*_pH7_ in the data set was 0.14 d^–1^ (half-life of about 5 days). The biodegradation was classified as “fast” when *k*_pH7_ was larger than the median, otherwise, it was classified as “slow”. The compounds were clustered into four groups showing consistently fast or slow biodegradation in more than 15 of the 18 river segments or showing variable but more frequently fast or slow biodegradation in different river segments.

Of the 47 compounds in the data set, 10 were classified as fast. Most of the fast compounds had a comparatively small spatial variability (stddev of log *k*_pH7_ usually lower than 0.4, Figure 3A). The inclusion of gap-filled data had minimal influence on the observed spatial pattern. Of the compounds classified as fast-degrading, bezafibrate and valsartan have been found to biodegrade relatively fast in both field and laboratory studies by others^14,35^ Seven compounds were classified as slowly biodegradable compounds. All of them had a very large spatial variability (stddev of log *k*_pH7_ > 0.63), which was strongly influenced by gap-filling (Figure 3A). Thirty compounds were classified as variable fast or variable slow. Most of them showed relatively large spatial variability (stddev of log *k*_pH7_ > 0.4 for 70% of the compounds, Figure 3A), and for half of them the spatial variability was influenced by gap-filling. Interestingly, when we included the results from the estuary site, ES_F_Pris_Salt, we found more compounds biodegraded slowly in this salty river segment compared with the freshwater rivers (Figures S10 and S11). ES_F_Pris_Salt was collected from a stream draining a lagoon through a sandbar about 100 m from the Mediterranean Sea. It had a salinity of about 28 ppt and was subject to frequent flooding with seawater and high erosion. The saltwater–freshwater mixing zones in estuarine areas are characterized by limited bacterial diversity,^36^ which has previously been suggested to be associated with slower biodegradation.^35^

Turning to the clustering of the river segments (the *x*-axis of Figure 3A), they are distributed according to decreasing log *k*_pH7_ moving from left to right. Of the different environmental variables, only TOC shows a consistent trend along this axis, decreasing from left to right (top of Figure 3A). This is consistent with the RDA analysis, which identified TOC as the most important explanatory variable (Figure 2). We further performed a Pearson correlation test for the median *k*_pH7_ (across all compounds) versus TOC and found a significant positive correlation between the two (*r* = 0.74, *P* < 0.001). A correlation between TOC and biodegradation rate has been proposed in a model of biodegradation^37^ and was also reported in a previous study of biodegradation in water-sediment systems.^14^ Some clustering within countries was found for CH, SE, and EL (Figure 3A). However, three river segments (DE_T_Contam, EL_G_Contam, and ES_C_Contam) were very clearly separated from other segments from the same country. Interestingly, the TOC for these river segments differed markedly from others in the same country. We did not observe cross-regional clustering of contaminated or pristine river segments (Figure 3A). For most of the highly concentrated compounds (>1 μg L^–1^), there was no correlation between their concentration in the surface water and *k*_pH7_ (Figure S12), which is in agreement with our previous observations.^5^ Only the much faster biodegradation of MET in SE_K_Contam was found to coincide with its much higher concentration.

In order to compare the spatial pattern of biodegradation rates of individual compounds, the values of log *k*_pH7_ were centered to the median of the 18 river segments (log *k*_pH7,centered_). The cluster analysis was repeated and the columns were grouped by country to explore whether country-specific biodegradation existed for some compound groups (Figure 3B). Interestingly, we found chemical class specific patterns of the spatial variability of log *k*_pH7,centered_. For instance, most sulfonamides (SMX and STZ) and carboxylic acids (BEZ, CLA, FUR, KET, MCPA, MEC, NAP, and VAL) clustered closely (Figure 3B). Some of these compound groups also showed country-specific biodegradability. For instance, sulfonamides usually biodegraded fastest in EL, and carboxylic acids usually biodegraded fastest in CH. In addition, we found that most of the compounds from the “variable_fast” cluster in Figure 3A usually had relatively fast biodegradation in CH or ES, and most of the compounds from the “variable_slow” cluster in Figure 3A biodegraded relatively fast in EL or DE (Figure 3B). Some compounds sharing the same functional group did not cluster closely (Figure 3B), e.g., phenylureas (CTU, DIU, ISO, and MXR). The complex relationship between different functional groups and biodegradation rates could be one possible explanation. Some compounds have multiple functional groups, and some functional groups are biodegradation promoters while some are inhibitors.^38^ For instance, many carboxylic acids are well biodegraded by oxidation or conjugation reactions, and the carboxylic acid group is considered to substantially improve biodegradation.^39−44^ CIL usually biodegraded faster than other carboxylic acids (Figure 3A) and was separated from other carboxylic acids in Figure 3B, which may be due to it being the only carboxylic acid with two carboxyl groups.

### Implications and Perspectives

Here, we measured the biodegradation of 97 compounds in 19 river segments across Europe, generating 1646 biodegradation rate constants. The variability between chemicals, evaluated as the stddev of the log biodegradation rate constant in a given river segment, was about a factor of 9 (median of all river segments). This was much larger than the spatial variability in rate constants for individual chemicals, which was about a factor of 3 (median of all chemicals). The spatial variability was greater than the seasonal variability reported in our earlier work (median stddev of about a factor of 2).^5^ We note that the spatial variability also contained a small element of seasonal variability (summer to midautumn). It was not possible to separate the effects of seasonal and temporal variability. The seasonal variability observed in our previous study was not systematic and varied from compound to compound,^5^ so we do not believe that it can readily be extrapolated to other locations. The spatial variability in valid rate constants among the 18 freshwater river segments was statistically significant for 98% of the compounds. The spatial variability, while considerable, might not be considered high for some applications like exposure modeling, where other parameters (e.g., regional-scale emission rates) often have comparable uncertainty.^4^ However, in other applications such as comparing simulation test results against bright-line regulatory thresholds, the observed spatial variability could present serious challenges.

That said, most if not all applications of biodegradation rate constants, be it exposure modeling or regulatory testing, would benefit from a better understanding of the spatial variability. We found that three factors, namely longitude, TOC, and particle size distribution of sediment, significantly explained the spatial variability of biodegradation rates. There is no clear and direct mechanistic link between these geographical and environmental properties and biodegradation rate constants that could serve as a foundation for a better understanding of spatial variability. Indeed, the causal links could be indirect and complex, such as particle size distribution influencing the depth and extent of hyporheic flow, which in turn influences the microbial biomass and species composition, which in turn influences the rate constant. However, the data at least indicate that we should strive to further elucidate the drivers of spatial variability. First, although TCC did not prove to be a useful descriptor of spatial variability, TOC had a significant correlation with the overall biodegradation rates. This indicates that there may be useful and easily measurable surrogates of active microbial biomass that may serve as predictors of spatial variability. Furthermore, similarities in the spatial variability of biodegradability were found for some compound groups sharing a given functional group. This indicates that there may be potential for group-specific benchmarking techniques to describe spatial variability.^45^ There might be other relevant variables determining the variability for individual chemicals that were not captured in this study, e.g., bacterial community composition and capabilities (functional genes, functional taxa, production of specific enzymes, etc.). Most likely, better pipelines for connecting data from molecular microbiology with the biodegradation rate constants could further improve our understanding of the spatiotemporal variation in the biodegradation rates of chemicals.

OECD 309 is the primary test for higher tier assessment of persistence under the European chemical legislation REACH. The test outcome is directly compared with the limit for half-life in water that is encoded in the regulation.^17^ The modified version of this test employed here shows high variability in test outcomes around the regulatory thresholds for P (40 days) and vP (60 days).^16^ The median stddev of rate constants of compounds that had a median half-life between 30 days and 70 days in European rivers was a factor of 5 (Figure 3A). Tests with water/sediment mixtures from multiple and diverse sites would be needed in order to constrain the uncertainty in the rate constant to a level satisfactory for regulatory purposes. To help overcome this problem, we suggest two possible strategies for further exploration:

1. Using easily measurable proxies for active microbial biomass (e.g., TOC) to preassess the overall biodegradation capacity of potential sampling sites, and then use a predefined window of the proxy as the site selection criterion.
2. Compounds that biodegrade fast usually show low spatial variability and could be tested at one site. Compounds whose test outcomes are close to the bright line of persistence criterion should be tested at more sites. This strategy could be further optimized once we better understand how spatiotemporal variability is related to chemical structure.

In addition, consistency between laboratory and field measurements is an important requirement for using laboratory tests for regulatory purposes. In the present study, the biodegradation rates were measured in laboratory tests. Several studies have found poor correlations between laboratory and field measurements of biodegradation rates,^46−48^ while others have found good correlations.^14,49^ Although we have endeavored to make the laboratory test more representative of conditions in the environment, establishing whether the spatial variability in biodegradation rates observed in these laboratory tests accurately reflects the situation in the field requires further study.

In this work, we have studied the biodegradation rate of chemicals in rivers. There are other aquatic systems where biodegradation can be an important factor affecting local exposure or global chemical fate such as wetlands, lakes, and the oceans. Our measurement in a brackish river segment provides a first indication that there could be significant differences in biodegradation rates between different classes of aquatic systems. This warrants further investigation.

## Supporting Information Available

Details on sampling sites, materials, methods, and data evaluation procedures, as well as graphs and tables containing complementary results (PDF)
Biodegradation kinetic parameters of quantified test compounds (XLSX)
