# Supplementary material for: Variability of Biodegradation Rates of Commercial Chemicals in Rivers in Different Regions of Europe
Source: Environ Sci Technol. 2024 Oct 28;58(45):20201–10. doi: 10.1021/acs.est.4c07410 (PMC11562712; doi:10.1021/acs.est.4c07410)
Supplement: Supplementary file 1 — es4c07410_si_001.pdf [file es4c07410_si_001.pdf]

Supporting Information

Variability of biodegradation rates of commercial chemicals in rivers in different regions of Europe

Run Tian,[1] Malte Posselt,[1] Kathrin Fenner,[2,3] Michael S. McLachlan[1]*

[1]Department of Environmental Science (ACES), Stockholm University, 10691 Stockholm, Sweden

[2]Eawag, Swiss Federal Institute of Aquatic Science and Technology, 8600 Dübendorf, Switzerland

[3]University of Zürich, Department of Chemistry, 8057 Zürich, Switzerland

*Corresponding author: run.tian@aces.su.se

Number of pages: 43

Number of tables: 5

Number of figures: 12

Table of Contents

S1. Characteristics of the sampling sites 5

S2. Additional information on chemicals used 9

S3. Biodegradation experiments 14

S4. Biodegradation kinetics of quantified test compounds 15

S5. Gap-filling 30

S6. Abiotic dissipation 31

S7. Environmental Parameters 34

S8. Statistical Results 36

List of Tables

Table S1. List of sampling sites 6
Table S2. Characteristics of the sampling sites 7
Table S3. List of test compounds 9
Table S4. List of internal standards. 13
Table S5. Ratio between dissipation rate constants in test treatments and the sorption controls (SC) for chemicals showing a significant $k_{observed}$ and dissipation in SC in at least one experiment 32

List of Figures

Figure S1. Locations of the sampling sites.. 5
Figure S2. Principal component analysis (PCA) of environmental factors measured at 19 different river segments.. 8
Figure S3. Biodegradation kinetics of the quantified test chemicals 29
Figure S4. Flowchart of the gap-filling process 30
Figure S5. Clustered heatmaps showing log k (d^{-1}) of compounds that had dissipation in the SC that strongly influenced the estimation of k (marked by *) within at least one given country 33
Figure S6. Trends of pH, dissolved oxygen (DO, mg L^{-1}), and EC (µS cm^{-1}) during each experiment... 35
Figure S7. Histograms displaying the standard deviation of log k (d^{-1}) and log k_{pH7} (d^{-1}) and the significance of the spatial variation (ANOVA) across all 18 river segments 37
Figure S8. Violin plots show the median, 25th, and 75th percentiles of the standard deviation (stddev) of log k (d^{-1}). Histograms show the percentage of compounds with significant differences (ANOVA, $P < 0.05$) across river segments. Results were derived from 18 river segments 38
Figure S9. Redundancy analysis (RDA) shows the relationship between log k (d^{-1}) and 10 environmental factors in 18 river segments in five European countries (SE, DE, CH, ES, EL). The bar chart presents the percentage of the variance explained (R^2, unbiased, Envfit) for each environmental factor 39
Figure S10. Clustered heatmap showing log k_{pH7} (d^{-1}) for 47 compounds in 19 river segments 40
Figure S11. Clustered heatmaps showing log k (d^{-1}) and log k_{pH7} (d^{-1}) of all compounds in 19 European river segments 41
Figure S12. Correlation between valid k_{pH7} (d^{-1}) and concentration of the compound in the river. 42

S1. Characteristics of the sampling sites

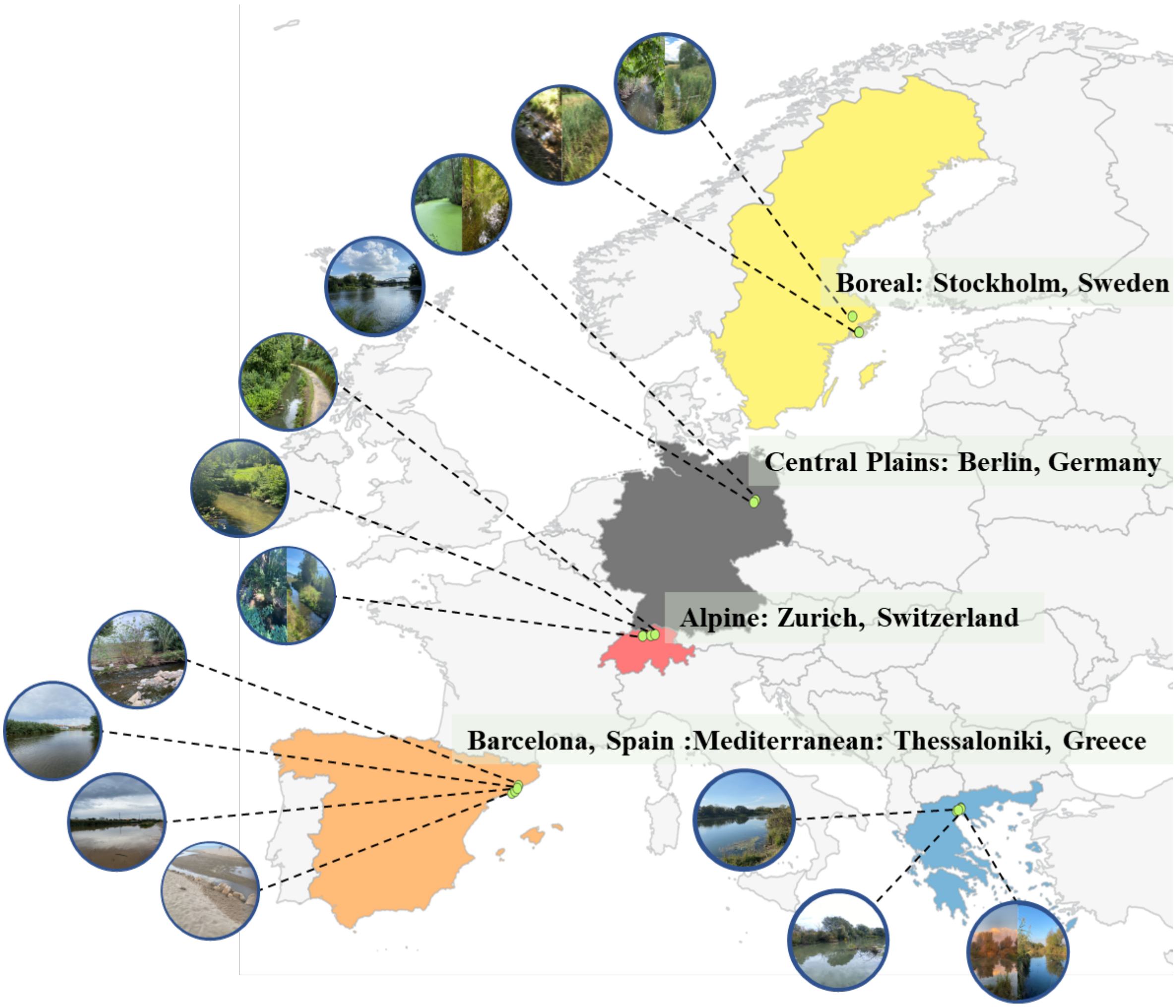

Figure S1. *Locations of the sampling sites. Map of sampling sites was plotted by QGIS (v.3.34.1).*

Table S1. *List of sampling sites. The abbreviations (Abbr.) of the river segments are used in Figure 3, Supplemental Dataset S1, and Supporting Information Tables S2, S5, and Figures S3, S6, S10-S12.*

Country	City	River	Status	Abbr.	Sampling Date	Location
Sweden	Stockholm	Vitsån	Pristine_Freshwater	SE_V_Pris	2022.08.01	~1000 m upstream of the Fors WWTP
Sweden	Stockholm	Vitsån	Contaminated	SE_V_Contam	2022.08.01	~700 m downstream of the Fors WWTP
Sweden	Stockholm	Knivstaån	Pristine_Freshwater	SE_K_Pris	2022.08.01	~500 m upstream of the Knivsta WWTP
Sweden	Stockholm	Knivstaån	Contaminated	SE_K_Contam	2022.08.01	~500 m downstream of the Knivsta WWTP
Germany	Berlin	Tegeler Fliess	Pristine_Freshwater	DE_T_Pris	2022.08.25	~50 m upstream of WWTP sluice gate
Germany	Berlin	Tegeler Fliess	Contaminated	DE_T_Contam	2022.08.25	~600 m downstream of WWTP sluice gate
Germany	Berlin	Havel	Contaminated	DE_H_Contam	2022.08.25	~2000 m downstream of the Ruhleben WWTP
Switzerland	Zurich	Uerke	Pristine_Freshwater	CH_U_Pris	2022.07.12	~2500 m upstream of the Kölliken WWTP
Switzerland	Zurich	Uerke	Contaminated	CH_U_Contam	2022.07.12	~500 m downstream of the Kölliken WWTP
Switzerland	Zurich	Reppisch	Contaminated	CH_R_Contam	2022.07.12	~1000 m downstream of the Birmensdorf WWTP
Switzerland	Zurich	Chriesbach	Contaminated	CH_C_Contam	2022.07.12	~500 m downstream of the Eawag WWTP
Spain	Barcelona	Fondo d'Aimaric	Pristine_Salty_Estuary	ES_F_Pris_Salt	2022.09.14	~100 m from the Mediterranean, pristine estuarine
Spain	Barcelona	Congost	Contaminated	ES_C_Contam	2022.09.14	~2000 m downstream of the Granollers WWTP
Spain	Barcelona	Besos	Contaminated	ES_B_Contam	2022.09.14	~3000 m downstream of the Besos WWTP
Spain	Barcelona	Llobregat	Contaminated	ES_L_Contam	2022.09.14	~7000 m downstream of the Llobregat WWTP
Greece	Thessaloniki	Gallikos_up	Pristine_Freshwater	EL_G_Pris	2022.10.28	~3000 m upstream of the Thessaloniki WWTP
Greece	Thessaloniki	Gallikos_down	Contaminated	EL_G_Contam	2022.10.28	~300 m downstream of the Thessaloniki WWTP
Greece	Thessaloniki	Axios	Contaminated	EL_A_Contam	2022.10.28	~2000 m downstream of urban agglomerations, located in agricultural production areas
Greece	Thessaloniki	Loudias	Contaminated	EL_L_Contam	2022.10.28	~10000 m downstream of the Loudias WWTP, located in agricultural production areas

Table S2. *Characteristics of the sampling sites (longitude, latitude; pH, dissolved oxygen (DO), electrical conductivity (EC), and temperature (Temp) of the water(W); total organic carbon (TOC) and particle size distribution (PSD, clay, sand, and silt content) of the sediment (S); total cell count (TCC) in W and S). Temp, pH, DO and EC were measured on-site.*

River	Longitude	Latitude	pH	DO (mg/L)	EC (µS/cm)	Temp (°C)	TOC (g/L)	TCC_W (cells per incubation bottle)	TCC_S (cells per Incubation bottle)	PSD (clay/silt/sand content, %)
SE_V_Pris	18.073	59.108	7.5	5.5	466	15.7	0.84	9.0E+09	9.9E+08	1 / 36 / 63
SE_V_Contam	18.098	59.102	7.7	8.4	568	18.9	0.69	1.2E+10	1.8E+09	1 / 42 / 58
SE_K_Pris	17.788	59.723	7.4	5.1	615	17.1	0.44	2.1E+09	2.4E+09	0 / 11 / 89
SE_K_Contam	17.792	59.716	7.3	6.7	683	19.4	2.05	1.8E+10	3.7E+09	1 / 33 / 66
DE_T_Pris	13.289	52.601	6.8	4.4	732	20.9	0.18	5.4E+09	2.3E+09	0 / 8 / 92
DE_T_Contam	13.282	52.598	6.8	7.1	829	23.0	0.94	2.1E+10	1.9E+09	0 / 14 / 86
DE_H_Contam	13.203	52.513	7.7	8.5	984	24.1	0.12	1.4E+10	3.5E+09	0 / 0 / 100
CH_U_Pris	8.047	47.364	8.4	9.8	491	14.5	0.17	4.5E+09	1.1E+09	0 / 0 / 100
CH_U_Contam	8.033	47.343	8.3	9.6	590	16.1	0.19	8.5E+09	1.2E+09	0 / 1 / 99
CH_R_Contam	8.413	47.368	8.2	10.7	809	17.9	0.23	9.1E+09	1.6E+09	0 / 1 / 99
CH_C_Contam	8.606	47.403	8.1	7.7	823	15.0	0.17	8.7E+09	1.3E+09	0 / 0 / 100
ES_F_Pris_Salt	1.956	41.265	8.1	7.6	42200	24.4	0.23	3.4E+09	1.5E+09	0 / 0 / 100
ES_C_Contam	2.254	41.552	8.1	6.9	1269	25.2	0.20	1.1E+10	2.8E+09	0 / 1/ 99
ES_B_Contam	2.203	41.448	7.8	4.3	1590	23.8	0.32	4.3E+10	2.1E+09	0 / 6 / 94
ES_L_Contam	2.099	41.337	7.8	4.7	1651	24.8	0.78	2.5E+10	2.3E+09	4 / 86 / 10
EL_G_Pris	22.826	40.689	8.1	8.3	768	15.7	0.85	7.5E+09	1.1E+09	0 / 44 / 56
EL_G_Contam	22.831	40.658	8.0	8.1	866	15.8	0.18	8.3E+09	2.2E+09	0 / 8 / 92
EL_A_Contam	22.712	40.605	8.3	9.4	410	17.1	0.63	9.8E+09	1.1E+09	1 / 58 / 41
EL_L_Contam	22.630	40.577	7.7	3.4	541	18.8	0.79	1.3E+10	1.3E+09	1 / 45 / 54

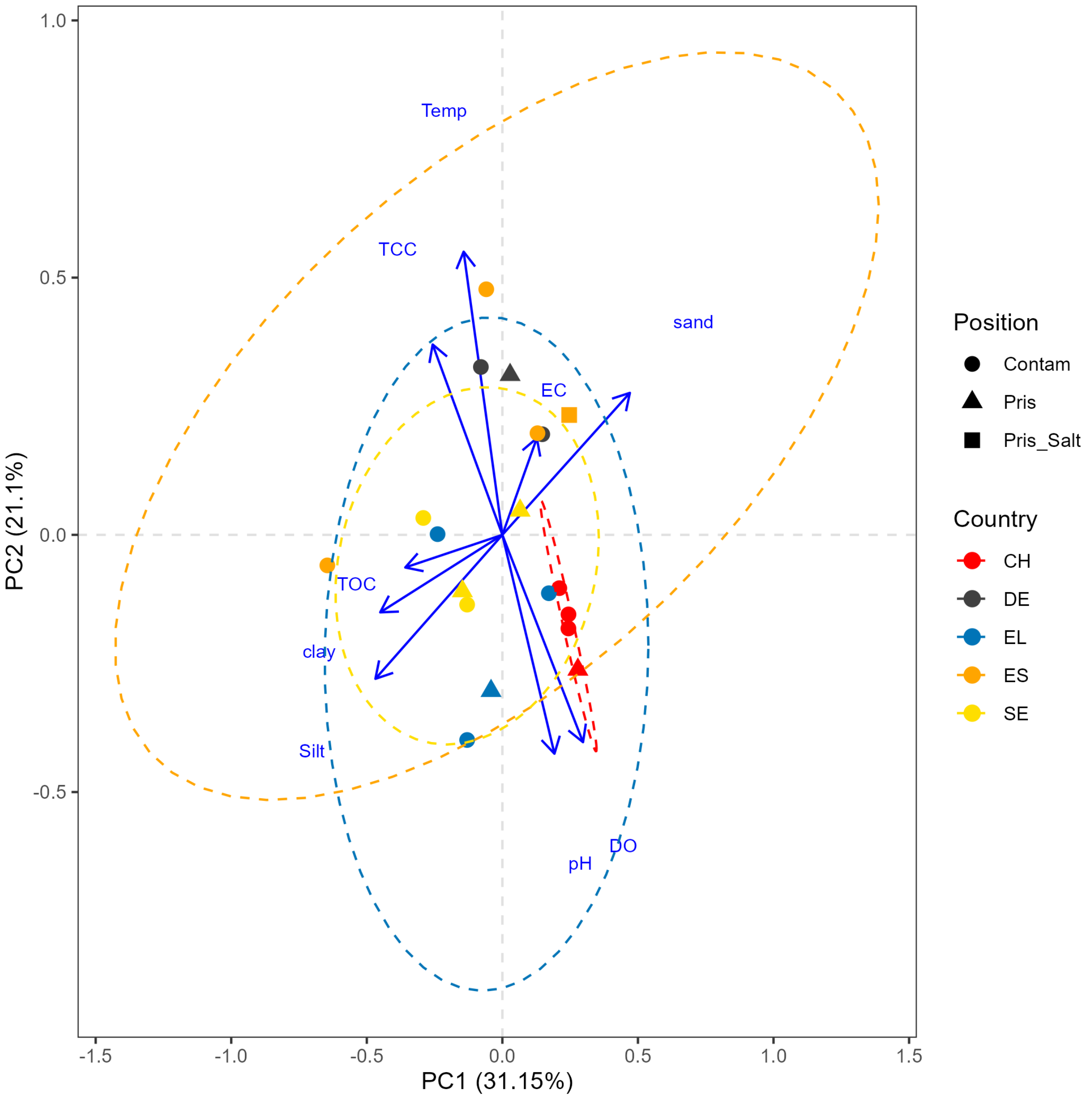

Figure S2. *Principal component analysis (PCA) of environmental factors measured at 19 different river segments, with the 95% confidence ellipses of each country. The environmental factors include Temp, pH, DO, and EC of water (W), TOC, and PSD (the content of clay, sand, and silt, %) of the sediment (S); TCC in the incubator (sum of TCC_S and TCC_W). These factors were measured in 19 European river segments (including 13 Contam: contaminated river segments, five Pris: pristine freshwater river segments, and one Pris_Salt: one pristine salty river segment).*

S2. Additional information on chemicals used

The test chemicals were purchased from Sigma-Aldrich (Steinheim, Germany) and Toronto Research Chemicals Inc. (North York, Canada), or were gifts from Unilever. D- and ^{13}C-labeled chemicals were purchased from Toronto Research Chemicals Inc. and CDN Isotopes (Pointe-Claire, Quebec, Canada) for use as internal standards. A working solution containing all test compounds was prepared at a concentration of 1.75 μg mL^{-1} in Milli-Q water. An internal standard solution containing all isotope-labeled standards was prepared at a concentration of 1 μg mL^{-1} in methanol. All the solutions were stored in the dark at -20 °C until use. LC/MS-grade methanol was purchased from VWR (Stockholm, Sweden). LC/MS-grade formic acid was purchased from Sigma-Aldrich. Sodium azide (NaN_3) was purchased from Sigma-Aldrich. Milli-Q water was produced using a Milli-Q Integral Water Purification System (Merck Millipore, Stockholm, Sweden).

Table S3. *List of test compounds. The abbreviations (Abbr.) of the compounds are used in Figures 3, Supplemental Dataset S1, Supporting Information Table S5, and Figures S3, S5, S7, S10-S12.*

	**Compounds**	**Abbr.**	**CAS Number**	**log *D***	**p*K*a[3]**
1	1-Stearoyl-rac-glycerol	1ST	123-94-4	2.6	-
2	2,6-Di-tert-butyl-4-methylphenol	2DI	128-37-0	5.07	11.6*[a]
3	3-((2-Ethylhexyl)oxy)propane-1,2-diol	3ET	70445-33-9	2.4	13.64*[a]
4	3-Methyl-4-(2,6,6-trimethyl-2-cyclohexen-1-yl)-3-buten-2-one	3ME	127-51-5	4.22	-
5	4-(4-Nitrobenzyl)-pyridine	4NP	1083-48-3	2.6	5.51*[b]
6	4-Chloro-3,5-dimethylphenol	4CD	88-04-0	2.83	9.7[b]
7	5-Methylbenzotriazole	5MB	136-85-6	1.69	8.74*[b]
8	Abacavir	ABA	136470-78-5	1.32	5.8*[b]
9	Acesulfame	ASF	33665-90-6	-2.77	2.0[a]
10	Acetamiprid	ATP	135410-20-7	1.06	0.7[a]
11	Alachlor	ALA	15972-60-8	2.99	1.20*[a]
12	Amisulpride	AMI	71675-85-9	-0.43	9.37[b]
13	Anastrozole	ANA	120511-73-1	2.68	1.4[b]
14	Atazanavir	ATA	198904-31-3	4.61	4.42*[b]
15	Atenolol	ATE	29122-68-7	-1.85	9.58[b]
16	Atrazine	ATR	1912-24-9	2.66	4.2[b]
17	Azoxystrobin	AZO	131860-33-8	3.54	1.94*[b]
18	Benzotriazole	BTZ	95-14-7	1.5	8.37[b]
19	Benzyl alcohol	BEA	100-51-6	4.85	15.4[a]
20	Benzyl salicylate	BES	118-58-1	4.01	8.11*[b]
21	Bezafibrate	BEZ	41859-67-0	-0.11	3.83*[a]
22	Bisoprolol	BIS	66722-44-9	0.12	9.67*[b]
23	Bromoxynil	BRO	1689-84-5	3.77	3.86[a]
24	C12 Isethionate	CIS	7381-01-3	-	-
25	Caffeine	CAF	58-08-2	0.28	-1.2[b]
26	Candesartan	CAN	139481-59-7	0.04	2.45[b]

27	Carbamazepine	CBZ	298-46-4	2.28	13.9[a]
28	Carbendazim	CAR	10605-21-7	1.51	4.29[b]
29	Chlorothiazide	CTZ	58-94-6	-0.21	4.29[a]
30	Chlorthalidone	CTD	77-36-1	0.41	6.85[a]
31	Chlortoluron	CTU	15545-48-9	2.48	14.43*[a]
32	Cilastatin	CIL	82009-34-5	-1.9	9.14*[b]
33	Ciprofloxacin	CIP	85721-33-1	-2.23	8.77*[b]
34	Citalopram	CIT	59729-33-8	1.27	9.78[b]
35	Climbazole	CLI	38083-17-9	3.32	6.49[b]
36	Clofibric acid	CLA	882-09-7	-0.88	-
37	Cocoamidopropyl betaine	COC	61789-40-0	0.93	-
38	Cyclamate	CYC	100-88-9	-3.51	1.7[b]
39	Decylamine	DEC	2016-57-1	1.07	10.64[b]
40	Dibenzepin	DIB	4498-32-2	1.24	8.23[b]
41	Dicamba	DAB	1918-00-9	1.24	1.97[b]
42	Diclofenac	DIC	15307-86-5	1.37	1.97[a]
43	Diflufenican	DIF	83164-33-4	4.09	9.03*[b]
44	Dimethenamid	DIM	87674-68-8	2.45	1.16[b]
45	Dioctyl Sulfocinate Sodium Salt	DIO	10041-19-7	1.01	-0.75*[a]
46	Diuron	DIU	330-54-1	0.13	-
47	Dodecyl sulfate sodium salt	DSS	151-21-3	1.6	-1.5[a]
48	Dodecylamine	DOD	124-22-1	1.86	10.63*[b]
49	Dodecyltrimethylammonium chloride	DOC	112-00-5	-	-
50	Ethofumesate	EFS	26225-79-6	2.14	-
51	Ethylene glycol butyl ether	EGB	111-76-2	0.77	14.42*[a]
52	Ethylhexyl methoxycinnamate	EMC	5466-77-3	5.28	-4.8*[b]
53	Ethylparaben	EPB	120-47-8	2.48	8.34[a]
54	Fenhexamid	FHX	126833-17-8	4.17	7.3[a]
55	Fenofibrate	FFE	49562-28-9	5.01	-4.9*[b]
56	Fipronil	FIP	120068-37-3	3.71	-5.86[b]
57	Flecainide	FLE	54143-55-4	1.01	9.3[b]
58	Fluconazole	FCZ	86386-73-4	0.7	1.76[b]
59	Fludioxonil	FDO	131341-86-1	2.57	14.10[a]
60	Flufenacet	FFA	142459-58-3	3.01	0.31*[a]
61	Fluoxetine	FXT	54910-89-3	1.75	9.8*[b]
62	Furosemide	FUR	54-31-9	-0.78	3.65[a]
63	Gabapentin	GAB	60142-96-3	-1.4	3.7[a]
64	Galaxolide	GAL	1222-05-5	5.93	-
65	Gemfibrozil	GEM	25812-30-0	1.58	4.5[a]
66	Hexylene glycol	HGL	107-41-5	0.23	15.10[a]
67	Homosalate	HOM	118-56-9	5.23	9.72*[a]
68	Hydrochlorothiazide	HCZ	58-93-5	-0.01	7.9[a]
69	Hydroxy bupropion	HBP	357399-43-0	1.93	-
70	Imidacloprid	IMI	138261-41-3	-0.29	1.56[b]
71	Iodopropynyl butylcarbamate	IBC	55406-53-6	3.2	14.4*[a]

72	Iprovalicarb	IPO	140923-17-7	3.29	11.41*[a]
73	Irbesartan	IRE	138402-11-6	1.24	5.85*[a]
74	Isoproturon	ISO	34123-59-6	2.45	15.06*[a]
75	Ketoprofen	KET	22071-15-4	0.06	3.98[a]
76	Lamotrigine	LAM	84057-84-1	1.68	5.7[b]
77	Levamisole	LAS	14769-73-4	0.25	6.98*[b]
78	Levetiracetam	LTC	102767-28-2	-0.74	-1.6*[b]
79	Lidocaine	LID	137-58-6	1.26	7.95[b]
80	Linezolid	LIN	165800-03-3	0.82	1.8[b]
81	Losartan	LOS	114798-26-4	1.29	5.5[a]
82	Loxynil	LOX	1689-83-4	1.27	3.96[a]
83	MCPA	MCPA	94-74-6	-1.09	3.13[a]
84	Mecoprop	MEC	7085-19-0	-0.65	3.21[a]
85	Mefenamic acid	MEF	61-68-7	2.04	4.2[a]
86	Metformin	MET	657-24-9	-3.36	12.4[b]
87	Methotrexate	MEX	59-05-2	-5.22	4.7[a]
88	Methyl 4-hydroxybenzoate	M4H	99-76-3	2.09	8.5[a]
89	Metolachlor	MLC	51218-45-2	3.22	1.45[a]
90	Metoprolol	MPL	51384-51-1	-0.25	9.56[b]
91	Metoxuron	MXR	19937-59-8	1.87	13.83[a]
92	N-(3-dimethylaminopropyl)-octadecanamide	NDO	7651-02-7	6.12	16.29*[a]
93	N,N-bis(2-hydroxyethyl)dodecanamide	NHD	120-40-1	3.95	14.13[a]
94	N,N-Bis(2-hydroxyethyl)tetradecanamide	NHT	7545-23-5	4.87	-
95	Naphthalene-2-carbonitrile	N2C	613-46-7	3.22	-
96	Naproxen	NAP	22204-53-1	0.45	4.15[a]
97	Neostigmine	NEO	59-99-4	-2.77	-
98	Nicotinamide	NIC	98-92-0	-0.37	3.35[b]
99	Novobiocin	NOV	303-81-1	-0.02	4.3[a]
100	Oxazepam	OXA	604-75-1	1.5	1.55[b]
101	Oxprenolol	OXP	6452-71-7	0.19	9.57[b]
102	Paracetamol	PAR	103-90-2	0.4	9.38[b]
103	Pargyline	PGL	555-57-7	2.31	8.05*[b]
104	Picoxystrobin	PIC	117428-22-5	3.84	-1.09*[a]
105	Propachlor	PPC	1918-16-7	2.28	0.30*[a]
106	Propranolol	PPN	525-66-6	1.15	9.53[b]
107	Propyl 4-hydroxybenzoate	P4H	94-13-3	2.81	8.5[a]
108	p-Toluenesulfonic acid	PTA	104-15-4	-3.36	-1.34[a]
109	Ranitidine	RAN	66357-35-5	-0.63	7.8*[b]
110	Rufinamide	RUF	106308-44-5	0.42	12.64[a]
111	Sodium decyl sulfate	SDS	142-87-0	-	-
112	Sorbitan monolaurate	SML	1338-39-2	4.02	-
113	Sotalol	SOT	3930-20-9	-1.63	9.76[a]
114	Sulfadimethoxine	SMX	122-11-2	-0.49	6.91*[a]

115	Sulfamethazine	SMT	57-68-1	0.79	7.59[a]
116	Sulfamethoxazole	SMZ	723-46-6	-0.56	6.16*[a]
117	Sulfamethoxypyridazine	SMP	80-35-3	-0.29	6.84*[a]
118	Sulfathiazole	STZ	72-14-0	0.03	7.2[a]
119	Tamsulosin	TAM	106133-20-4	0.77	9.28*[b]
120	Tartrazine	TAR	1934-21-0	-10.17	-
121	Terbutryn	TBR	886-50-0	1.38	4.30[b]
122	Tetradecylamine	TAL	2016-42-4	3.05	10.62[a]
123	Tramadol	TRA	27203-92-5	0.52	9.41[b]
124	Triethyl citrate	TRC	77-93-0	1.09	11.82[a]
125	Trimethoprim	TMP	738-70-5	-1.15	7.12[b]
126	Trinexapac-ethyl	TAE	95266-40-3	-1.76	4.7[a]
127	Valsartan	VAL	137862-53-4	-0.89	3.6[a]
128	Venlafaxine	VEN	93413-69-5	1.43	9.5[b]
129	Zolpidem	ZOL	82626-48-0	3.06	5.65[b]

[1] It was difficult to distinguish 5-methylbenzotriazole from its isomers. Since the feature with retention time and mass spectrum in all 4 seasonal experiments was similar to that in calibration standards, the feature was assumed to be 5-methylbenzotriazole for subsequent comparisons.
[2] log D_{OW} was obtained from ChemSpider.
[3] p*K*a was obtained from PubChem, and the values with an asterisk were predicted values obtained from DrugBank and ChemBK. [a] acid pKa, [b] base pKa.

Table S4. *List of internal standards.*

	**Compounds**
1	2-hydroxy-Ibuprofen-d6
2	acesulfame-d4
3	acetaminophen-d4
4	atenolol-d7
5	atorvastatin-d5
6	bezafibrate-d4
7	caffeine-d9
8	climbazole-d4
9	carbamazepine-d8
10	clofibric acid-d4
11	conitine-(methyl-d3)
12	fluconazole-d4
13	gabapentin-d6
14	gemfibrozil-d6
15	glimepiride-d5
16	irbesartan-d6
17	ketoprofen-^{13}C, d3
18	MCPA-d6
19	mecoprop-d3
20	metformin-d6
21	metoprolol acid-d5
22	metoprolol-d7
23	naproxen-d3
24	oxazepam-d5
25	pravastatin-d3
26	propranolol-d7
27	sulfamethoxazole-d4
28	tramadol-d6
29	triethyl-d15-phosphate
30	valsartan-d3
31	venlafaxine-d6
32	zolpidem-d6

S3. Biodegradation experiments

The experimental setup followed the protocol of an OECD 309 test with slight modifications, which we have previously shown to provide better environmental relevance than the standard OECD 309 test.[1] Sampling was carried out in 19 river segments located in five European countries (section S1). Water samples were collected 20 cm below the surface and sediment was collected by taking the top 3-5 cm layer with a sediment core sampler. Multiple sediment samples were transferred into two separate two-liter amber glass bottles and mixed. All the samples were transported in insulated containers from the sampling sites to the lab. The incubations were started within 24 hours of sampling. Sediment was sieved to 2 mm and homogenized, 17.5 g of sieved sediment was added to 350 mL river water (50 g wet solid L^{-1}), and kept in suspension with an orbital shaker (KS 501 digital, IKA, about 150 rpm). The incubators were sealed with parafilm to minimize changes in water pH caused by the outgassing of CO_2 from the oversaturated river water. All experiments were carried out in the dark at river water temperature (20.5 ± 3.5 °C). The variations in temperature in rivers (surface water, field) were about 1-3 °C within each country and were considered to be within the expected range of temperature fluctuation during the day. Therefore, all experiments for a given country were conducted in the same temperature-controlled room simultaneously (SE: 19°C, DE: 24°C; CH: 17°C; ES: 24°C, EL: 19°C).

Test incubations (three replicates), sorption controls (SC, sterilized sediment-water mixtures for distinguishing biodegradation from sorption, two replicates), and a hydrolysis control (sterilized river water for distinguishing biodegradation and sorption from hydrolysis) were spiked with an aqueous mixture of 129 compounds (including pharmaceuticals, industrial chemicals, agrochemicals, food additives, and cosmetics) to a concentration of 1 μg L^{-1} each. All abiotic controls were sterilized with 0.1% sodium azide. The incubators were shaken for 10 minutes before the start of the experiment to ensure complete mixing. Dissipation of the test compounds was monitored by analyzing filtered (0.45 μm PTFE filters) subsamples taken from the water phase of each vessel after 0, 2, 5, 9, 18 hours, 1, 2, 4, 7, and 10 days. During incubation, aqueous pH, temperature, and EC were measured manually on each sampling day in each incubator, and DO was measured on a daily basis. The particle size distribution (PSD, including the content of clay, silt, and sand) of sieved and homogenized sediment was measured by Mastersizer 3000 (Malvern). Total organic carbon (TOC) was measured in sieved and homogenized sediment by Eurofins using the loss-on-ignition method. Cell density measurements were conducted in the sieved and homogenized sediment and river water prior to filling the incubator. Measurements were conducted on a BD Accuri C6 Flow Cytometer (BD, Belgium). Sampling and sample preparation for cell density analysis were based on our previous study.[2] Briefly, 5 mL river water or 5 g sieved sediment prior to addition to the incubator was transferred into a 20 mL amber glass vial containing 5 mL of 2% paraformaldehyde solution buffered with 0.1% pyrophosphate. All samples were stored at 4 °C in the dark until analysis. The measured cell density was used to calculate the total cell count (TCC) in the incubator.

S4. Biodegradation kinetics of quantified test compounds

The estimate of biodegradation rate constants was carried out following Tian et al.[2] Briefly, the peak area (A) at each time point was normalized to the compound's peak area at t=0 (A_0). The natural logarithm of the normalized peak area derived from SC was subtracted from that in the test treatment at each time point, and the resulting data were used to quantify the $k_{observed}$. About 5% of estimated $k_{observed}$ (91 out of 1672) displayed a significant increase in concentration over time ($P < 0.05$). In all but 11 of these cases, $k_{observed}$ was not significantly different from zero if no sorption correction was done. We concluded that the positive slopes were likely due to artifacts in the SC for chemicals with very low attenuation rates, and we treated these results as invalid $k_{observed}$. The 11 cases with significant increases in concentration both before and after SC correction were excluded from the dataset. To minimize the impact of possible fluctuations during the first incubation day on the regression analysis, time points from the first day were not used for estimating $k_{observed}$ unless the compounds degraded rapidly to below the LOQ within one day.

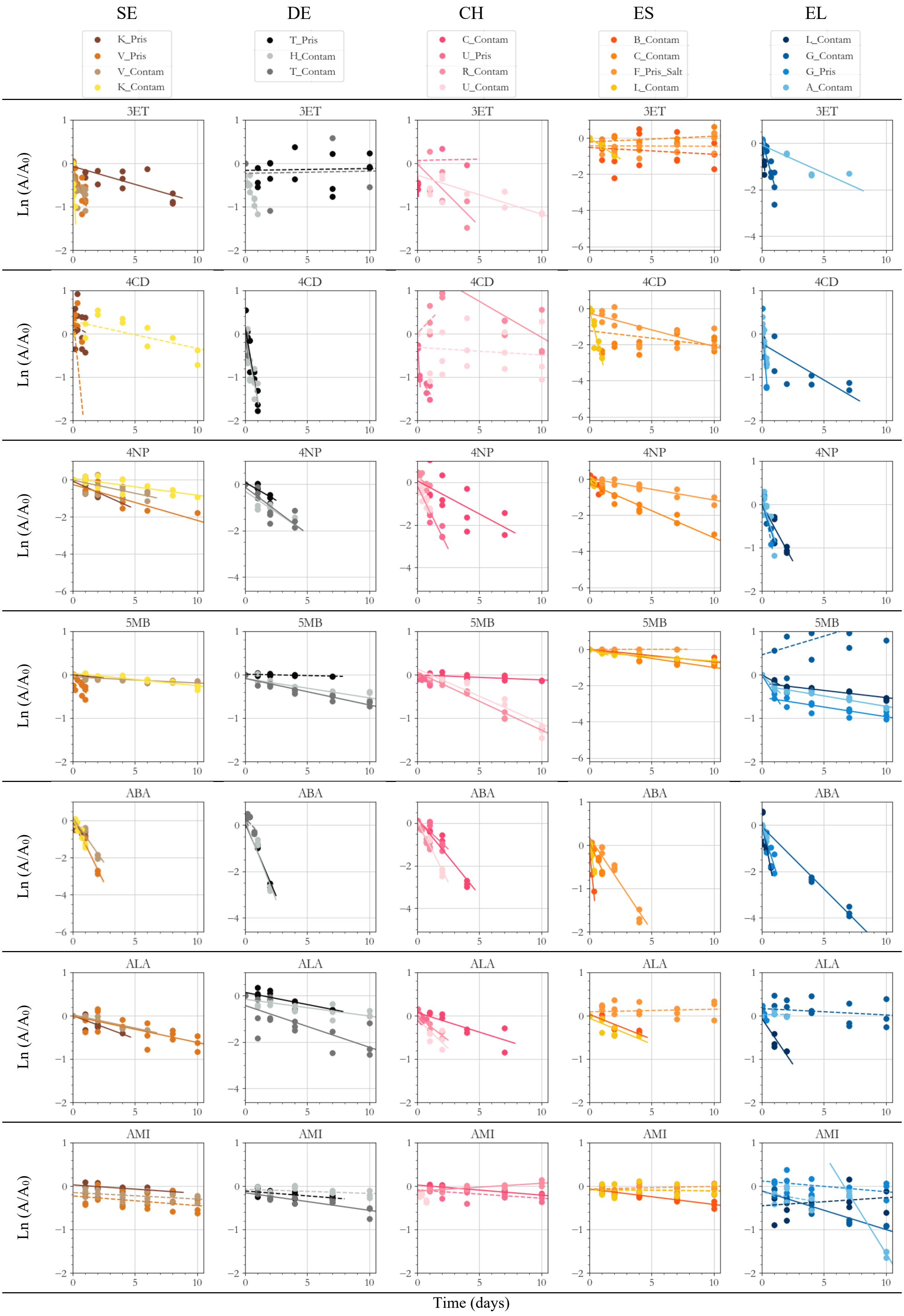
SE
DE
CH
ES
EL
K_Pris
V_Pris
V_Contam
K_Contam
T_Pris
H_Contam
T_Contam
C_Contam
U_Pris
R_Contam
U_Contam
B_Contam
C_Contam
F_Pris_Salt
L_Contam
L_Contam
G_Contam
G_Pris
A_Contam
3ET
4CD
4NP
5MB
ABA
ALA
AMI
Ln (A/A0)
Time (days)

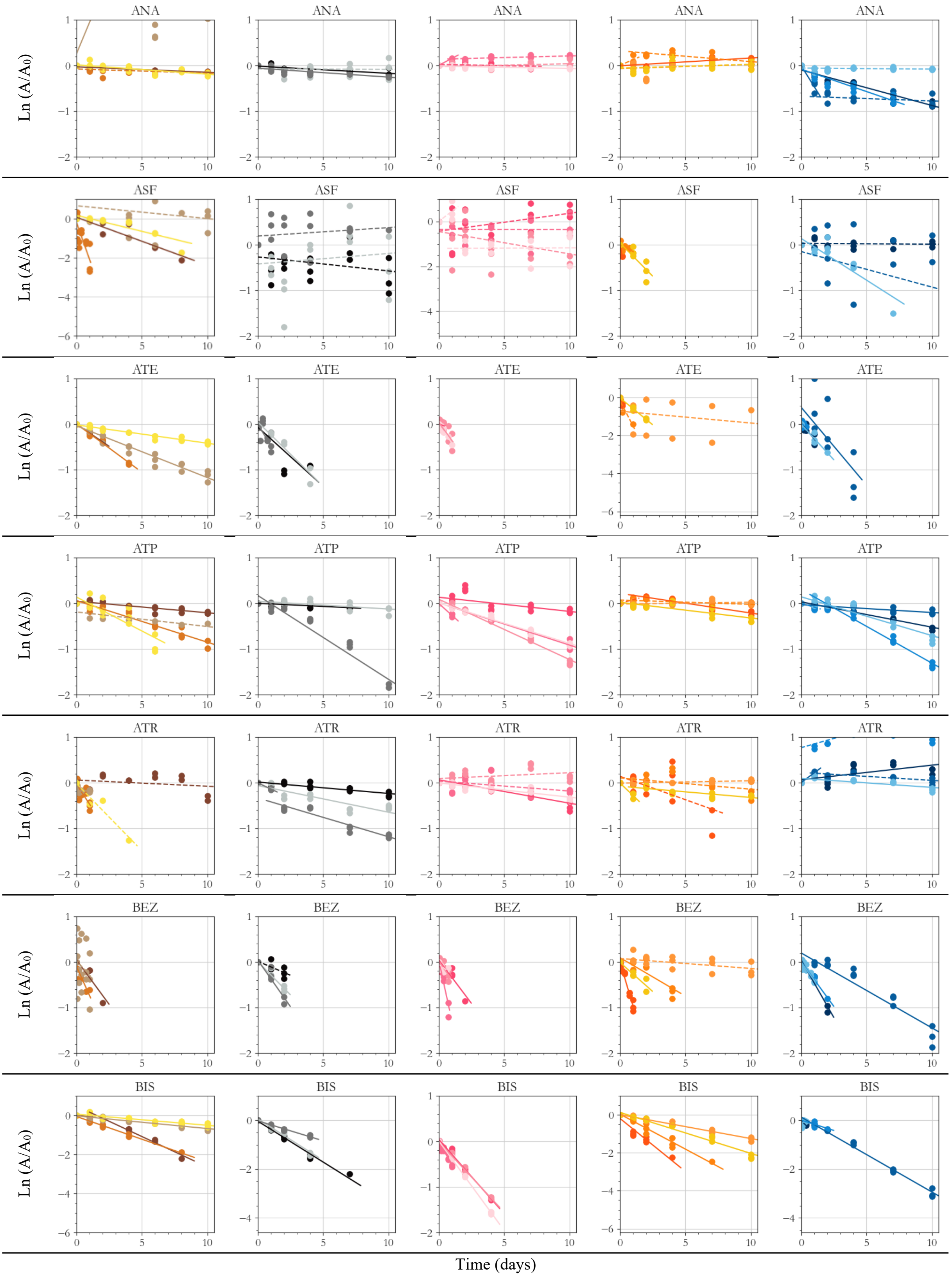
ANA
ASF
ATE
ATP
ATR
BEZ
BIS
Ln (A/A0)
Time (days)

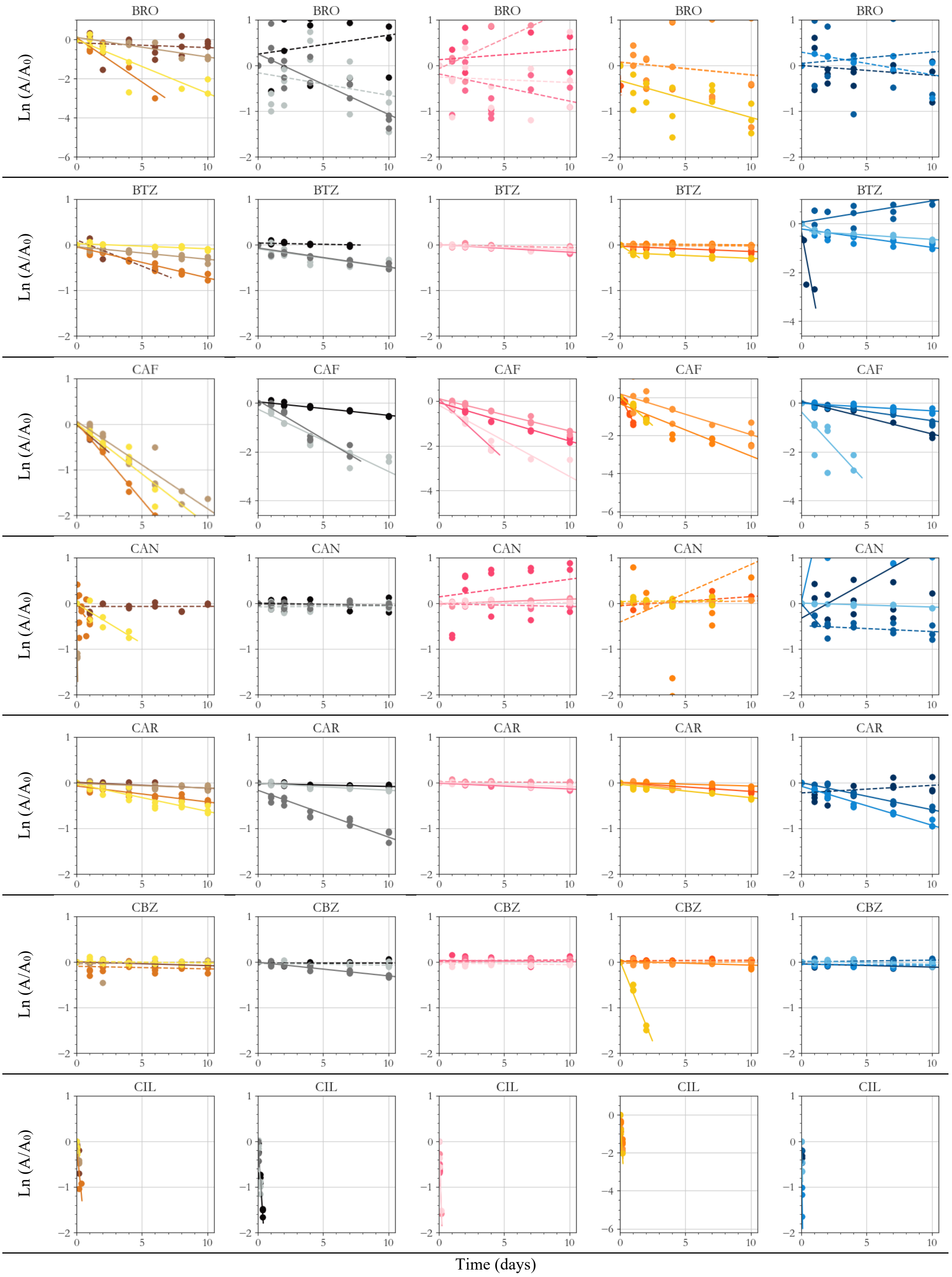

BRO
BTZ
CAF
CAN
CAR
CBZ
CIL
Ln (A/A0)
Time (days)

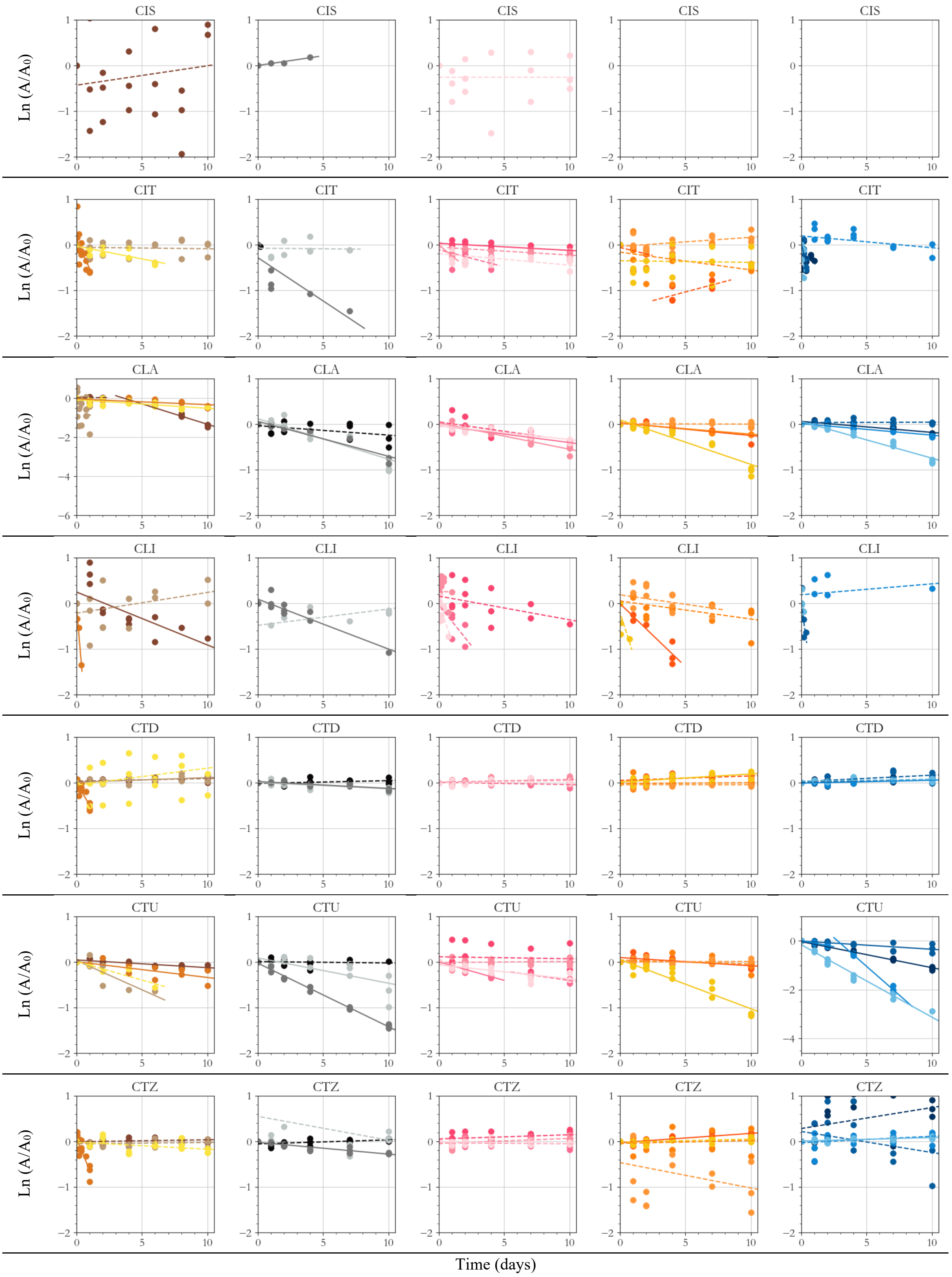
CIS
CIT
CLA
CLI
CTD
CTU
CTZ
Ln (A/A0)
Time (days)

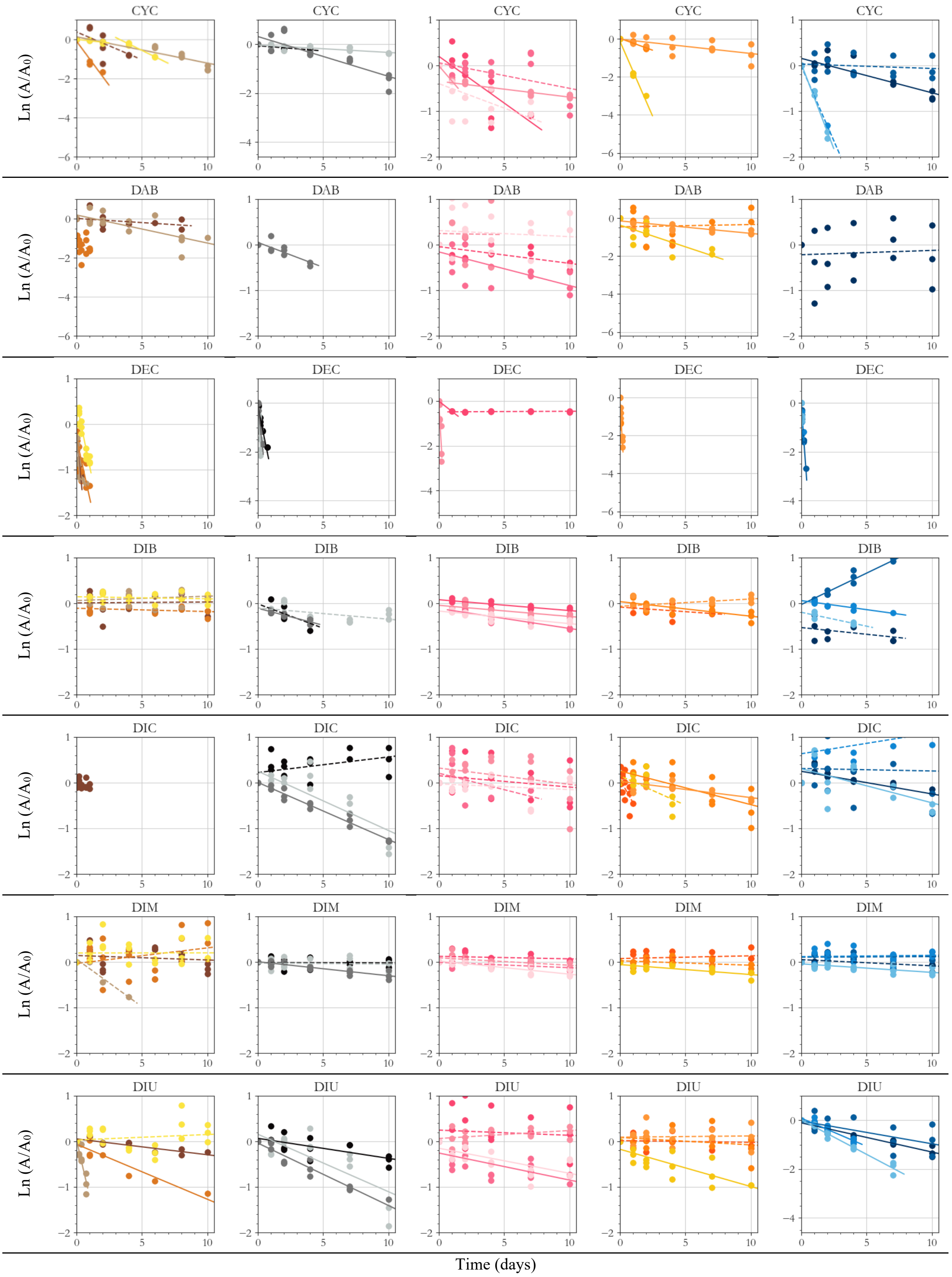

CYC
DAB
DEC
DIB
DIC
DIM
DIU
Ln (A/A0)
Time (days)

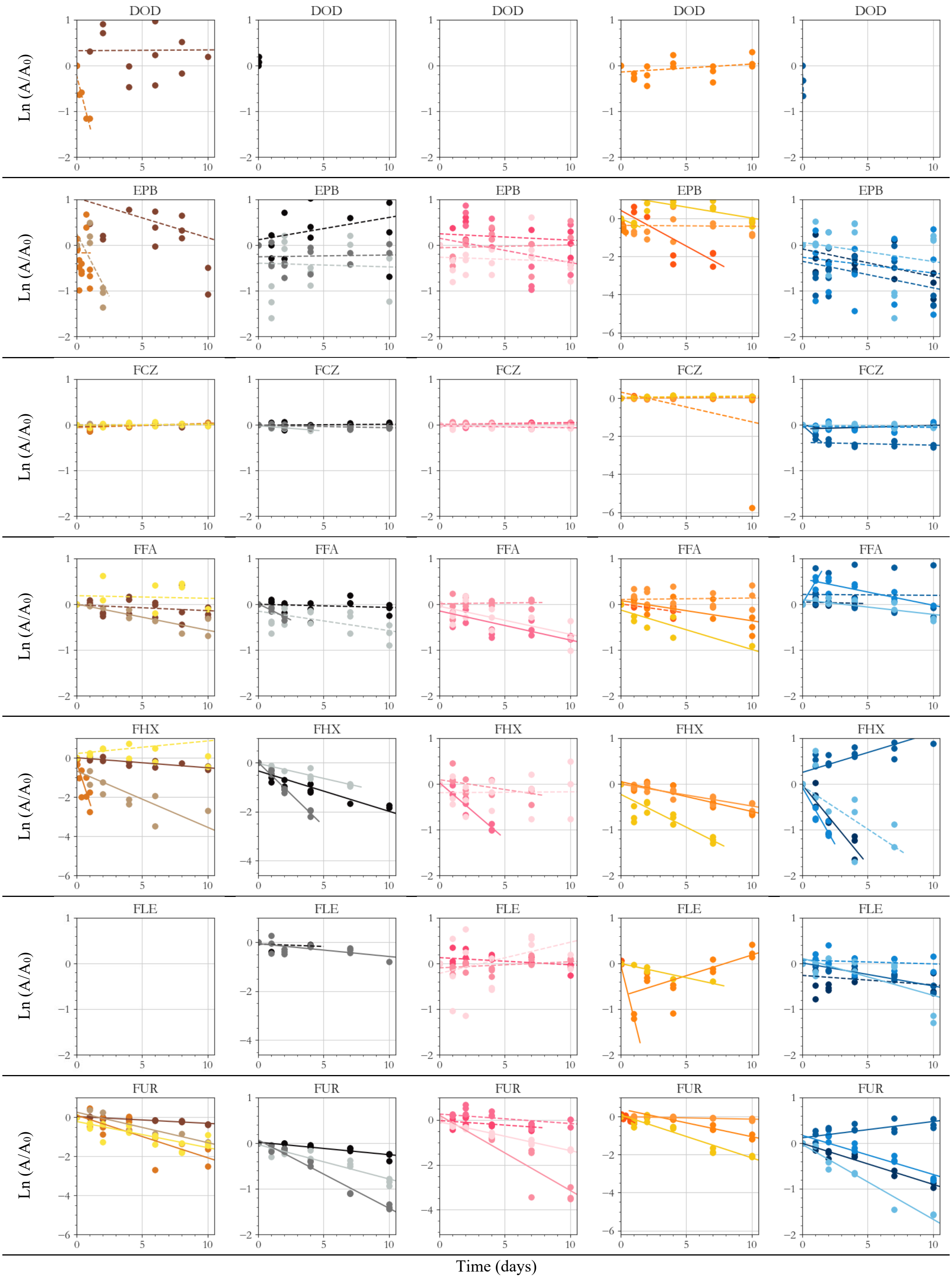

DOD
EPB
FCZ
FFA
FHX
FLE
FUR
Ln (A/A0)
Time (days)

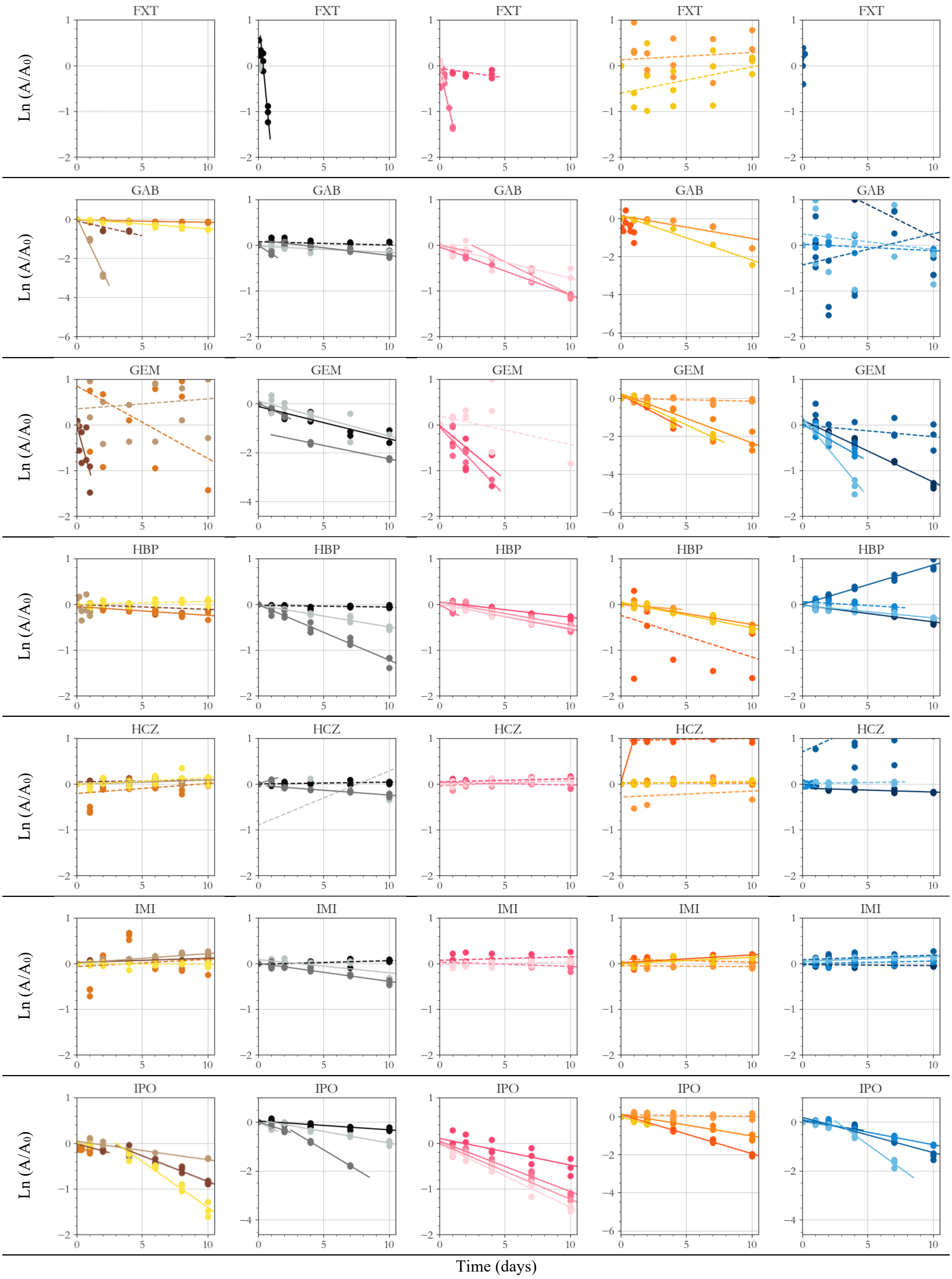
FXT
GAB
GEM
HBP
HCZ
IMI
IPO
Ln (A/A0)
Time (days)

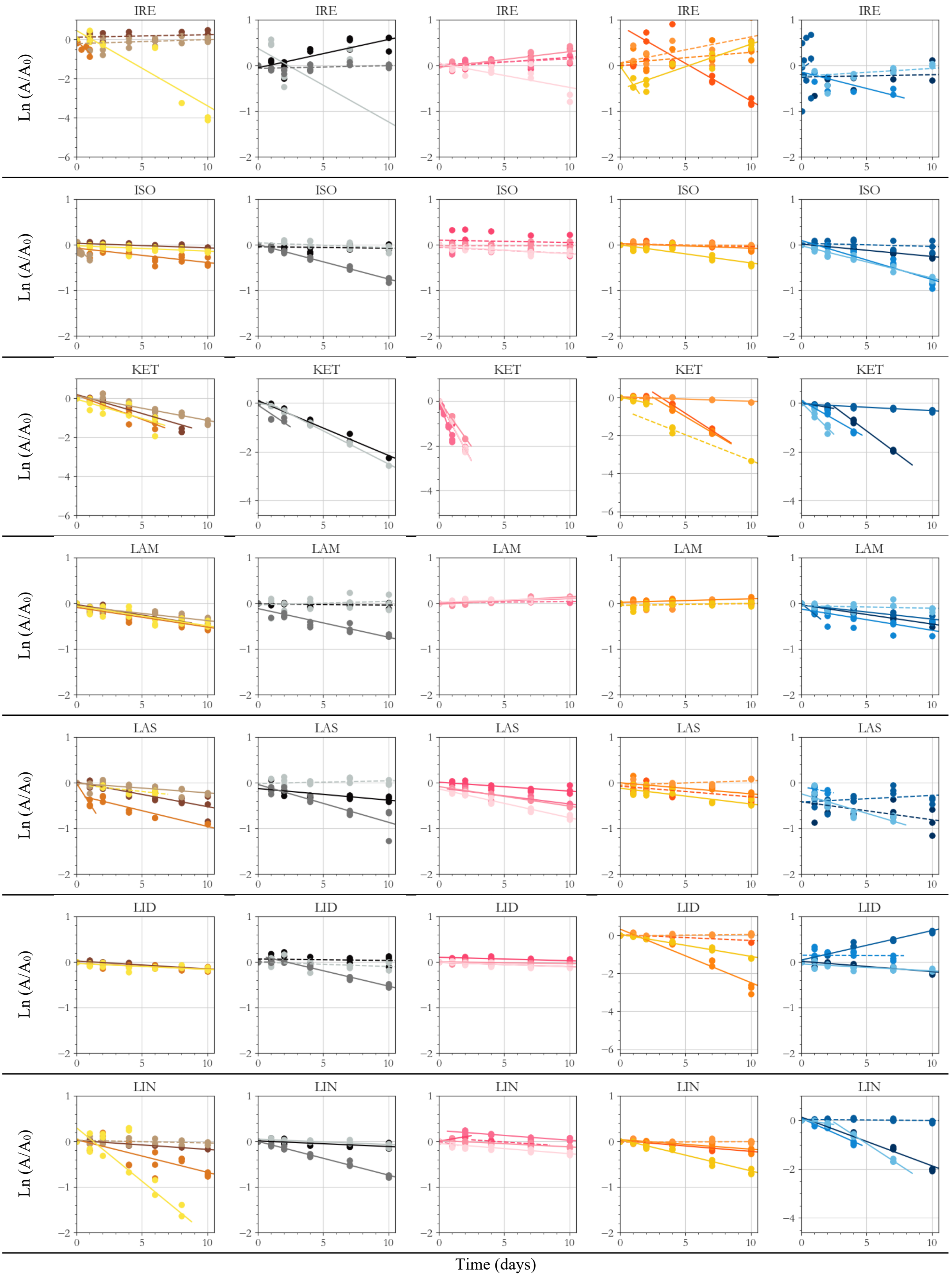

IRE
ISO
KET
LAM
LAS
LID
LIN
Ln (A/A0)
Time (days)

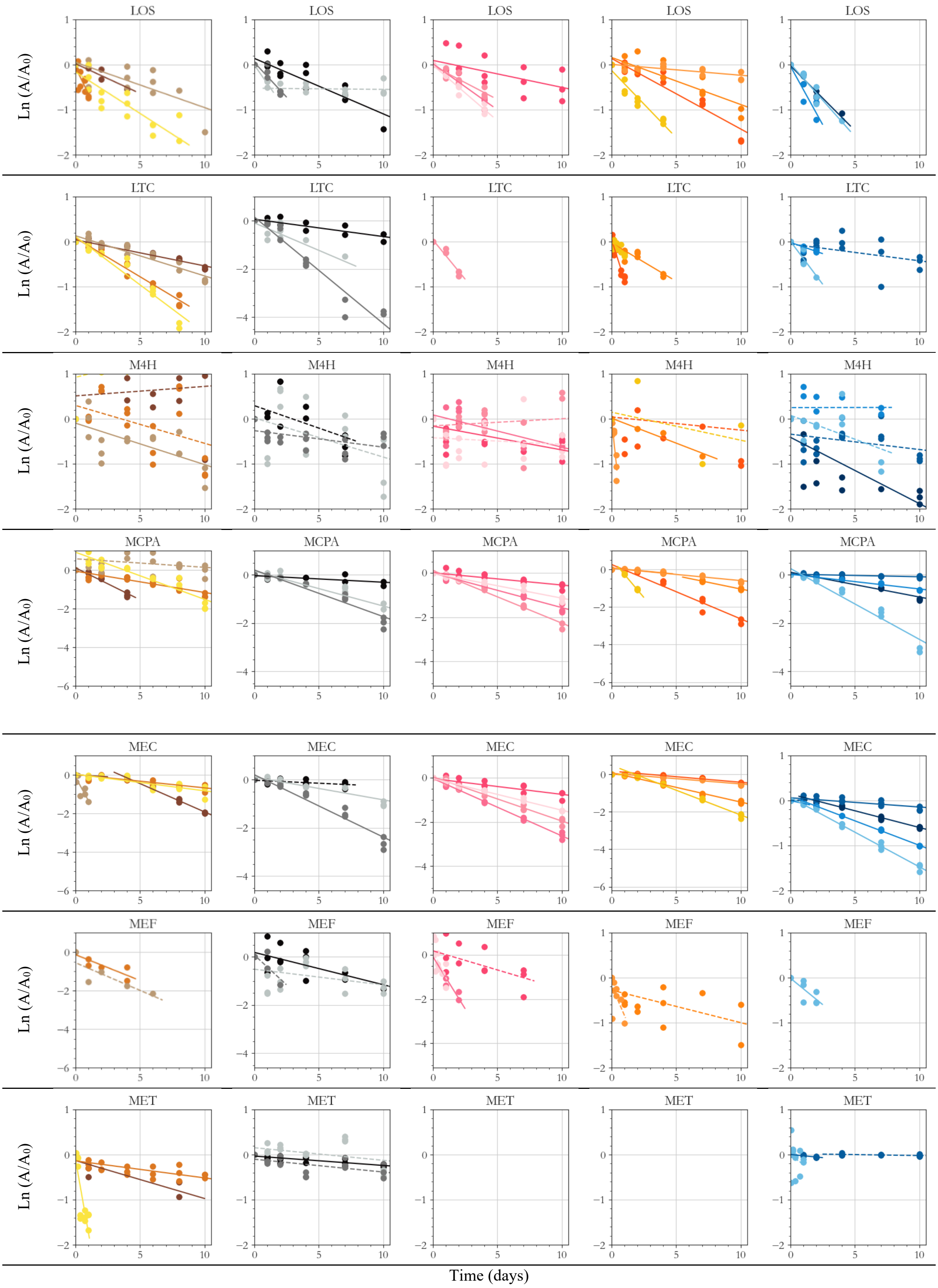
LOS
LTC
M4H
MCPA
MEC
MEF
MET
Ln (A/A0)
Time (days)

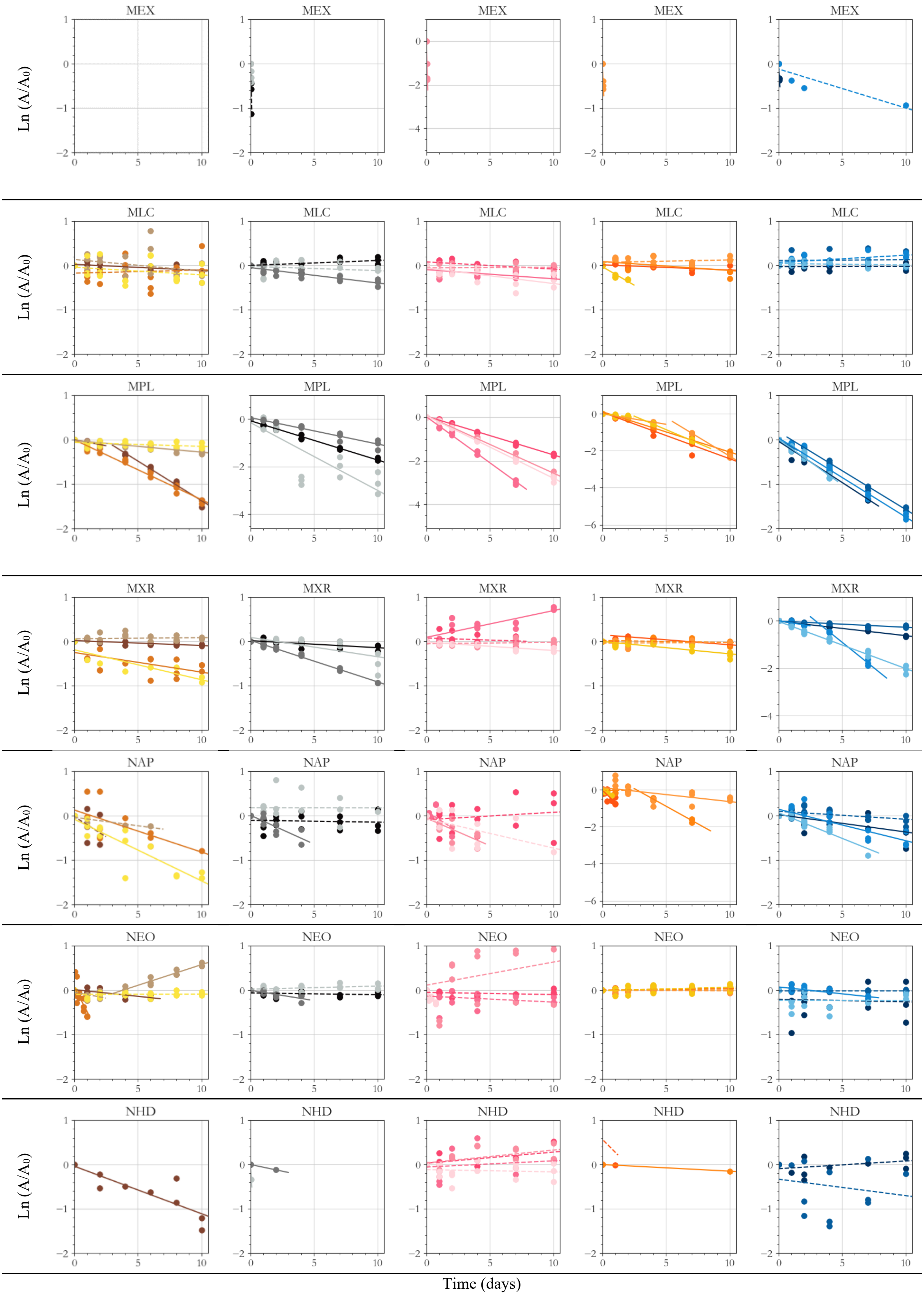
MEX
MLC
MPL
MXR
NAP
NEO
NHD
Ln (A/A0)
Time (days)

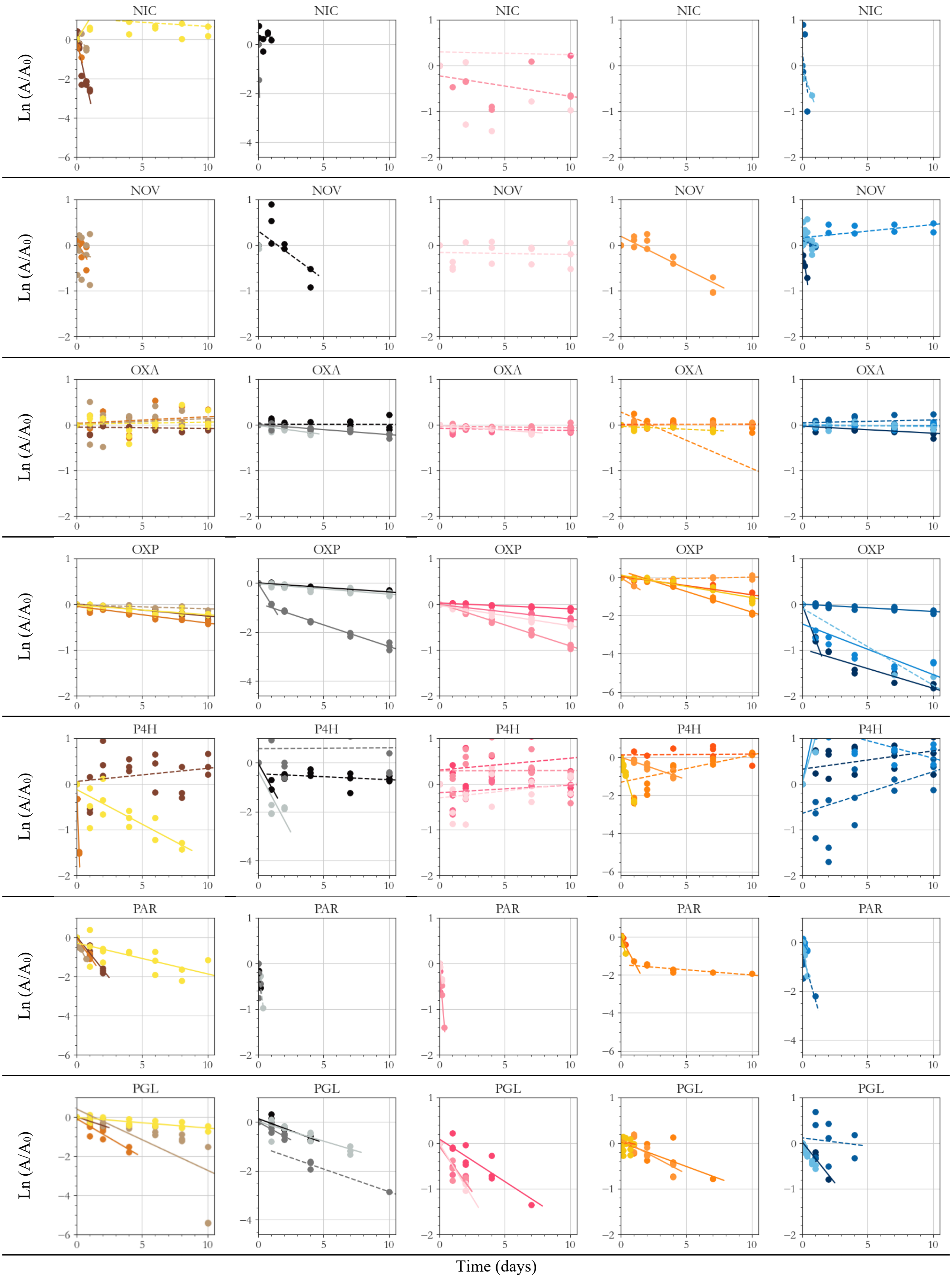
NIC
NOV
OXA
OXP
P4H
PAR
PGL
Ln (A/A0)
Time (days)

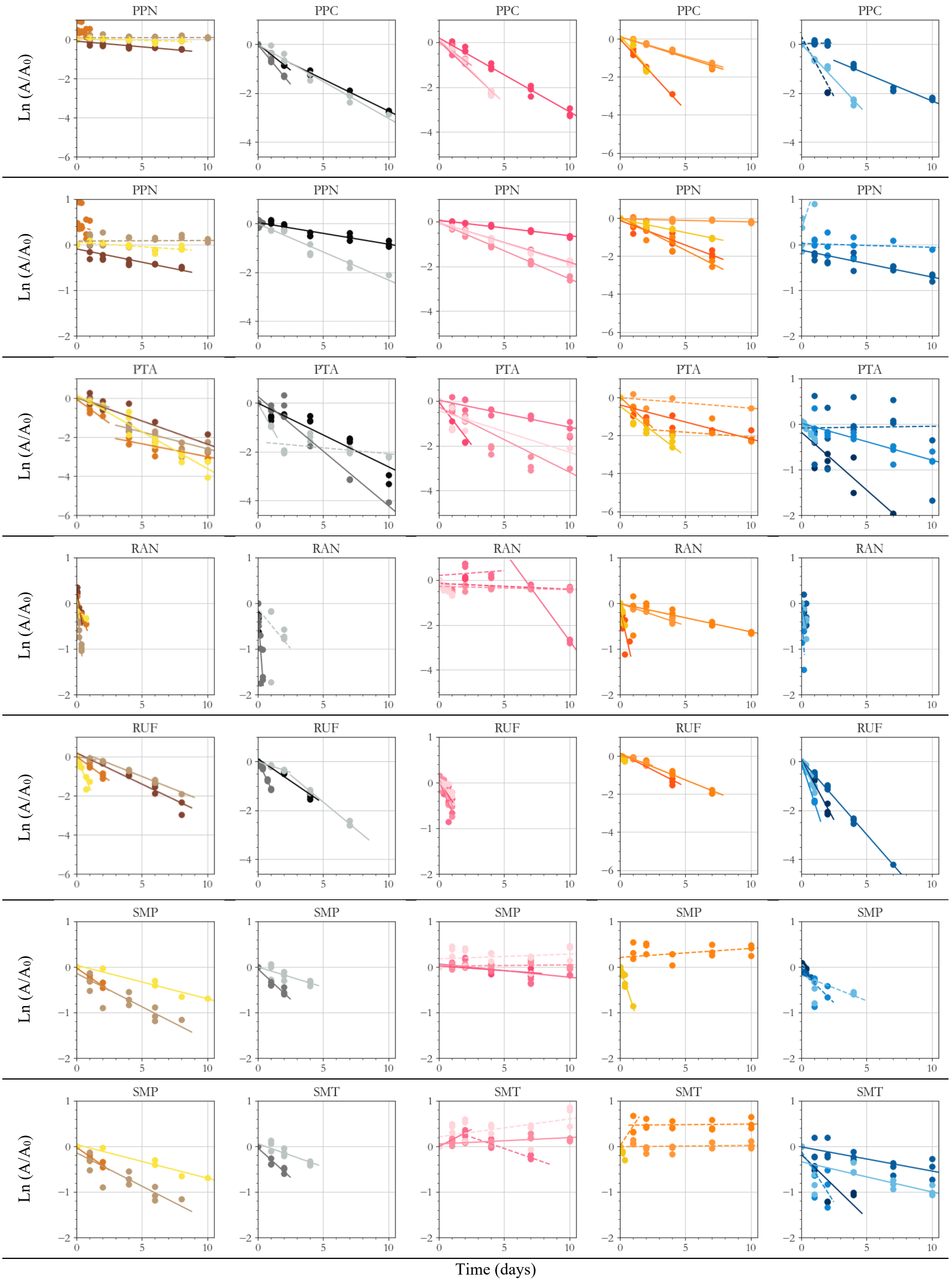

PPN
PPC
PPC
PPC
PPC
PPN
PPN
PPN
PPN
PPN
PTA
PTA
PTA
PTA
PTA
RAN
RAN
RAN
RAN
RAN
RUF
RUF
RUF
RUF
RUF
SMP
SMP
SMP
SMP
SMP
SMP
SMT
SMT
SMT
SMT
Ln (A/A0)
Time (days)

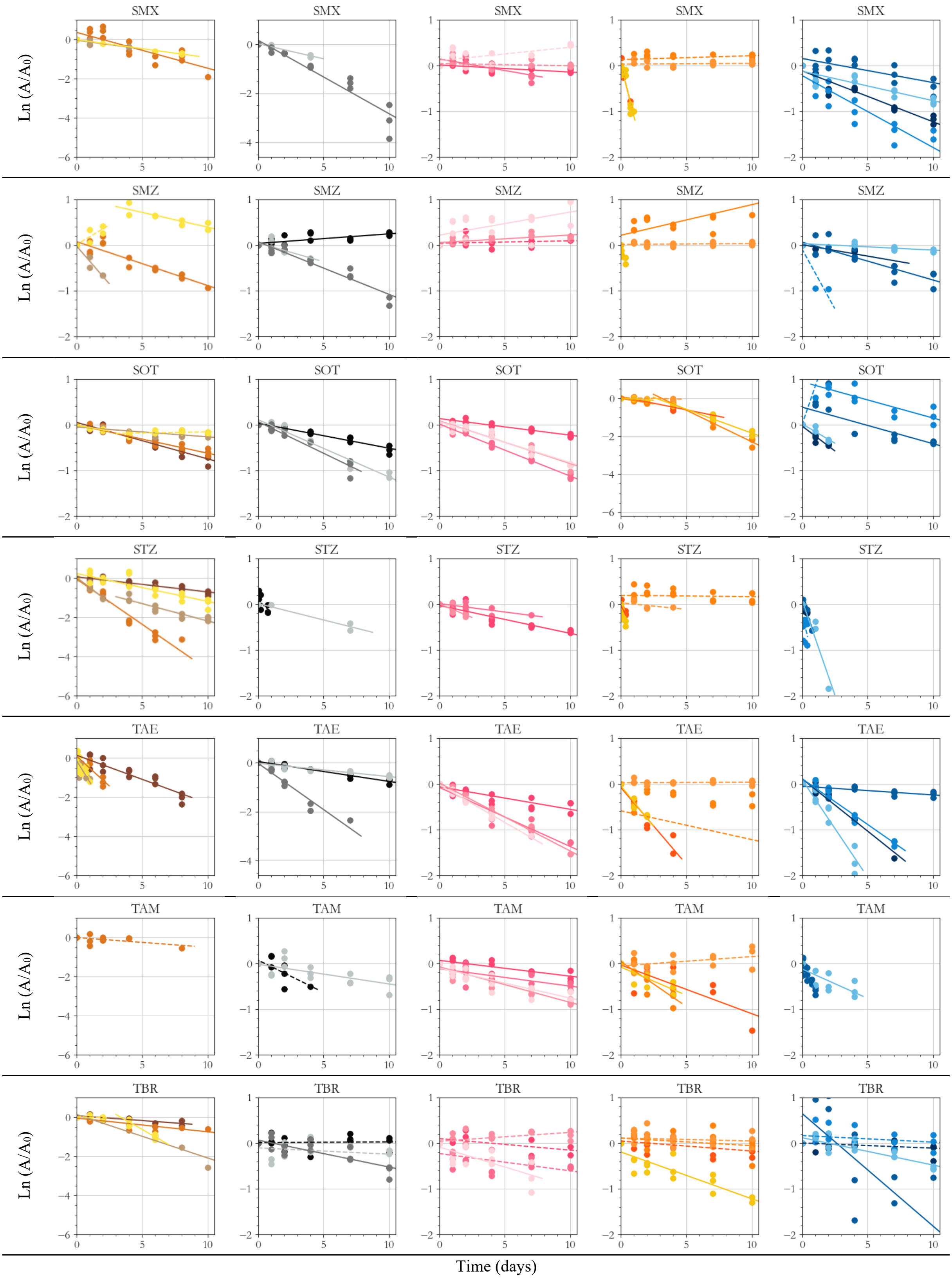

SMX
SMZ
SOT
STZ
TAE
TAM
TBR
Ln (A/A0)
Time (days)

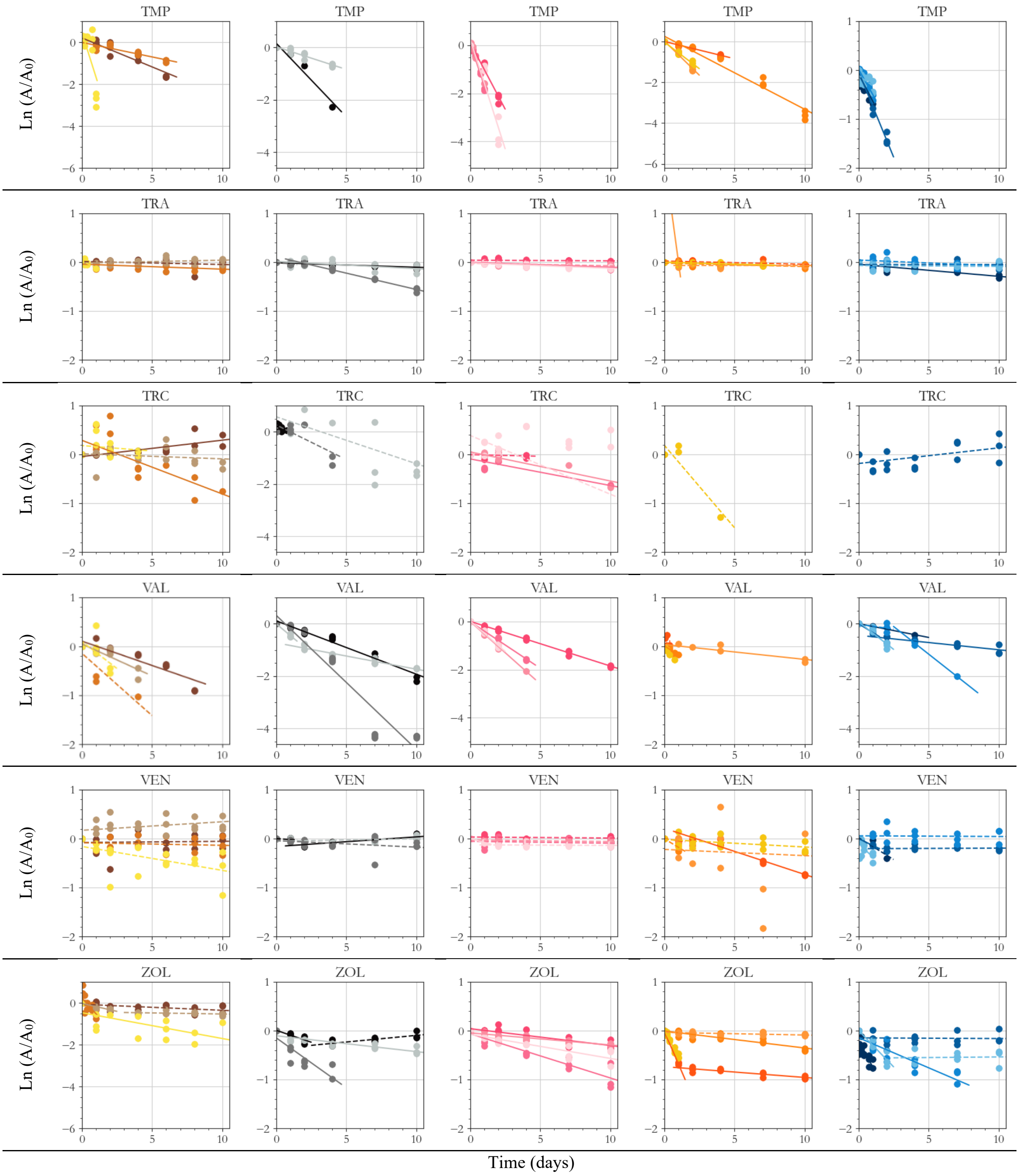

Figure S3. *Biodegradation kinetics of the quantified test chemicals. A dashed line indicates that the 99% confidence interval of the slope intersected zero. Each plot on a line shows the results for a different country, while the different lines in each plot represent the different river segments in that country. For elaboration of the country and river segment name abbreviations see Table S1. For elaboration of the compound name abbreviations see Table S3. Data for SE was from our previous study.*[2] *All data used to estimate biodegradation kinetics were SC corrected (without* f_{Dis} *correction).*

S5. Gap-filling

The estimated initial $k_{observed}$ was considered valid when it was based on at least five data points and significantly different from zero ($P < 0.05$). When no valid $k_{observed}$ could be calculated for a given compound (i.e., when one of these two conditions was not satisfied), we used a gap-filling approach employing the following decision chain to ensure that the gap-filled values were consistent with the observations (Figure S4):

1. if the 99% confidence interval of estimated $k_{observed}$ (invalid) contains the median valid $k_{observed}$ (for the same compound, all other river segments), replace with median valid kobserved (same compound, all other river segments), otherwise
2. if the 99% confidence interval of estimated $k_{observed}$ (invalid) contains the minimum valid $k_{observed}$ (for the same compound, all other river segments), replace with minimum valid $k_{observed}$ (same compound, all other river segments), otherwise
3. gap fill with the minimum valid $k_{observed}$ (all compounds, all river segments).

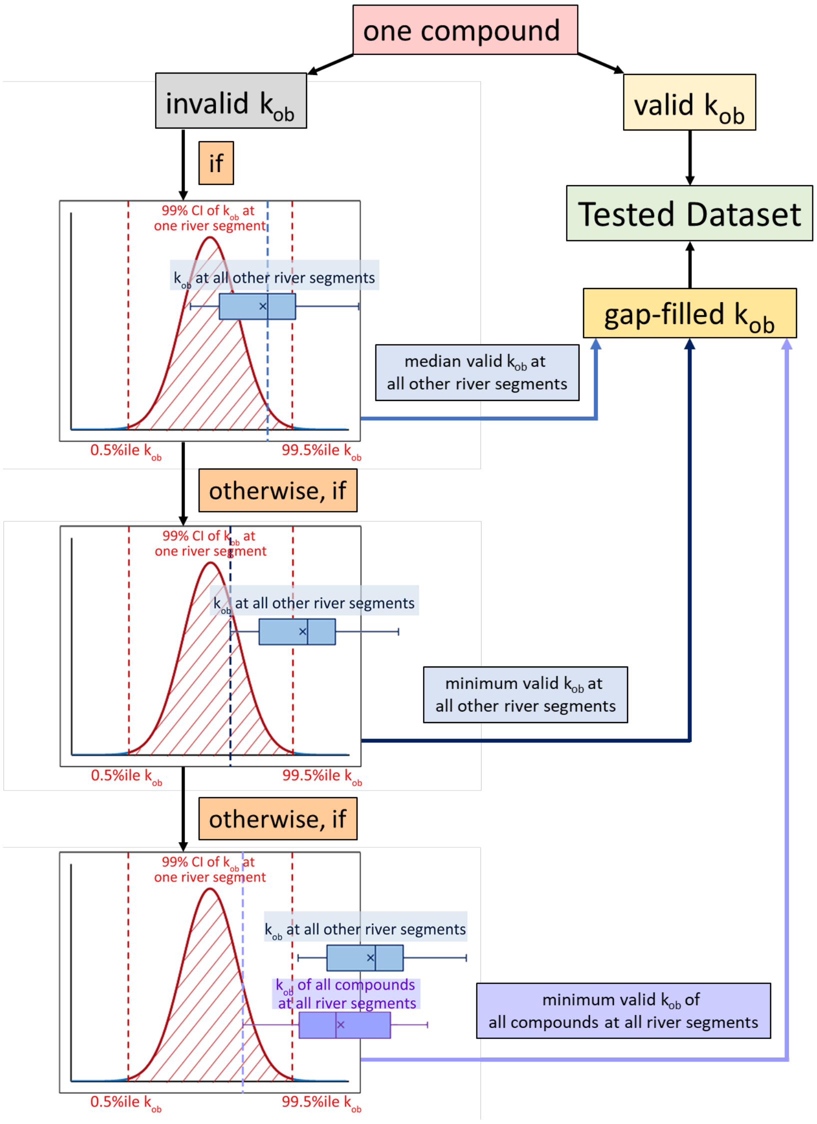

Figure S4. *Flowchart of the gap-filling process.* $k_{observed}$ *is denoted as* k_{ob} *in the plot. The boxplots show the range of the valid* k_{ob} *across given river segments.*

S6. Abiotic dissipation

The abiotic controls were meant to distinguish dissipation via biodegradation from dissipation via abiotic attenuation (hydrolysis and sorption). Six chemicals showed dissipation in the hydrolysis control (ethylparaben, methyl 4-hydroxybenzoate, nicotinamide, propyl 4-hydroxybenzoate, triethyl citrate, and irbesartan). Before the start of the experiment, 13 out of 97 quantified compounds (amisulpride, citalopram, climbazole, decylamine, dibenzepin, flecainide, fluoxetine, levamisole, propranolol, ranitidine, tamsulosin, trimethoprim, and venlafaxine) were significantly sorbed to the sediment (higher than 2-fold difference in peak area between hydrolysis control and SC) in at least 25% of the river segments. All of them were bases. When the content of clay and silt was < 20%, 70% of those compounds had a significant moderate correlation ($R^2 > 0.5$, $P < 0.05$) between the content of clay and silt and the fraction of the compound that was dissolved (f_{Dis}). However, when the content of clay and silt was > 20%, there was no correlation between it and f_{Dis}. 80% of these cases had a f_{Dis} lower than 0.5, and at low values of f_{Dis}, the dependence of f_{Dis} on the quantity of sorbent was weaker.

In the SC experiments, between 2 and 19 out of 97 compounds showed a first-order dissipation with a slope significantly different from zero. The ratio between the dissipation rate constants in the test treatments and SC is shown in Table S5. Out of 1646 cases, 90% had a dissipation rate constant in SC that was more than 2.5 times smaller than the dissipation rate constant in the test treatments. Overall, dissipation in the SC had a small effect on the experimental results. Amisulpride, dibenzepin, and levamisole had significant dissipation in SC both before the experiment and during the first day of the experiment in about 60% of the river segments. They also dissipated after the first day of the experiment but at a much slower rate compared to during the first day. The initial strong sorption led to a biphasic dissipation curve in the test treatments in some experiments, so for better comparison between all 19 river segments we used data measured after the first day to estimate $k_{observed}$ for these three compounds.

Faster dissipation in the SC compared to the hydrolysis control might be due to: i) abiotic reactions facilitated by the presence of the sediment; ii) sorption; and iii) residual biodegradation as a result of incomplete sterilization. In the first two cases, an SC correction of the test treatments is indicated, while in the third case, an SC correction should not be conducted. We could not ascertain which of these three phenomena was responsible for the dissipation in our experiments. Our decision to correct all of the test treatment data for dissipation in SC may have resulted in an underestimation of k in a few cases if the dissipation in SC was caused by phenomenon iii). 5-Methylbenzotriazole, amisulpride, atenolol, citalopram, dibenzepin, levamisole, rufinamide, and tramadol had dissipation in SC that strongly influenced the estimation of k within at least one country. The consequences for the evaluation of the spatial variation in biodegradation rates are shown in Figure S5.

Table S5. *Ratio between dissipation rate constants in test treatments and the sorption controls (SC) for chemicals showing a significant $k_{observed}$ and dissipation in SC in at least one experiment. Values marked in red indicate that the ratio was less than 2.5. Empty cells indicate that no significant dissipation was observed in the SC. For elaboration of the country and river segment name abbreviations see Table S1. For elaboration of the compound name abbreviations see Table S3. Data for SE was from our previous study.*[2]

Country	SE				DE			CH				ES				EL			
River	V_ Pris	V_ Contam	K_ Pris	K_ Contam	T_ Pris	T_ Contam	H_ Contam	U_ Pris	U_ Contam	R_ Contam	C_ Contam	F_ Pris_Salt	C_ Contam	B_ Contam	L_ Contam	G_ Pris	G_ Contam	A_ Contam	L_ Contam
5MB	7.1	1.6	1.6		2.2	1.2	2.2	22.1		18.6	2.8		21.3	6.0	13.6	10.0	1.0	7.3	4.8
ABA	13.8	9.4	5.2	8.7	8.4	1.5	7.6		5.8	8.1	8.7	5.0	3.3	5.8	2.2		1.4	3.0	
AMI			1.1		1.6	2.1	1.3	1.6	10.1		1.8						1.2	2.1	
ATE	3.4	3.0		1.8	2.2	2.2	2.6	2.0	1.2	1.2	1.1			1.7	2.9		3.5	1.7	2.1
ATP		4.2		4.5		2.0		18.9	7.5	14.7					3.7		2.0		4.3
BTZ	3.0	2.6				1.3	2.2						1.0	1.7	4.2	3.1	1.0	8.9	
CIL	42.4	42.2		70.7	10.6	15.1	12.2	20.4	13.6	35.3	28.2	21.6	9.2	1.0	6.7	1.0	8.2	13.0	2.9
CIT	7.8				1.1	1.2	1.0	1.0	1.0	1.3	1.0			3.4			3.4		
DEC	7.9	20.6	4.9	10.0		3.0	3.7			3.0		1.0	1.0				4.3	5.1	
DIB	1.6				2.0	2.5	1.9	2.5	2.0	2.0	1.7		2.4	1.7		1.7		1.9	3.2
FHX						9.8				1.6									10.1
HBP						2.5	3.1	17.8	7.6		1.6		2.2		2.2	1.4		1.9	2.0
HCZ					1.0			1.0											
IMI						1.7													
IRE					1.0	1.0	1.7	1.0	1.9		1.0				4.8	1.3			
LAS		2.3			1.6	1.9	1.0	3.4	3.9	8.1	2.2		2.4	1.7	1.3	3.3		2.6	1.9
LID		1.6				1.0		2.1		1.9					10.2		1.0	1.6	1.2
LIN	4.3					1.6		2.2	2.2	2.1				3.4	4.7	12.4	1.0	16.1	11.0
OXA						1.1	3.4	1.0	3.5						1.4				1.1
OXP		1.2	1.2		1.9		3.2	3.7	5.2	11.6	2.4			16.9	4.0		1.4		
P4H							6.3	1.0	1.0	1.0	1.0	1.8	3.5		1.9		1.0	1.0	1.0
PGL	11.7					3.7	5.2	14.9								1.0	1.5		3.0
PPC	6.5	4.3				3.2		9.3	6.9	2.9	2.4	2.6		5.1	2.8		4.2	6.1	1.7

PPN					1.2	1.0	5.2	5.1	1.5	8.9	1.4			9.9	1.3	1.0	1.7		
RAN	1.2	2.6	10.3	1.4	2.0	1.8		2.2	1.9	5.0	7.0	3.4	1.2	3.1	2.1	2.6		1.9	2.1
RUF	3.8	4.4	8.4	2.6	4.5	4.4	10.3	1.3	1.2	1.4	1.3	1.8	2.9	2.9	1.6	3.6	14.8	2.8	3.8
SMP						2.2		1.6							46.8		1.9		
SMT						9.6		1.8									2.2		
SMX								2.8											9.0
SMZ																	1.7		
STZ	9.5	21.4						1.8		4.4				17.3			6.8	38.1	5.6
TAE				8.1	3.9	6.6	3.3	3.0	3.4	3.3	2.2			4.5	3.1	5.0	1.8	7.6	4.8
TMP					24.7		15.1	60.4	56.1	57.2	42.7	12.7	54.9		14.8	12.2	3.8	5.3	11.1
TRA			1.1			2.2									1.1	1.0	1.0	1.0	1.9
TRC		1.1		1.1		2.1		1.2	1.0	1.3		1.0	1.6		1.7				
VEN									1.2								1.4		6.0
ZOL					2.9	4.9	10.6	10.8		3.4					19.7	3.9		6.0	13.2

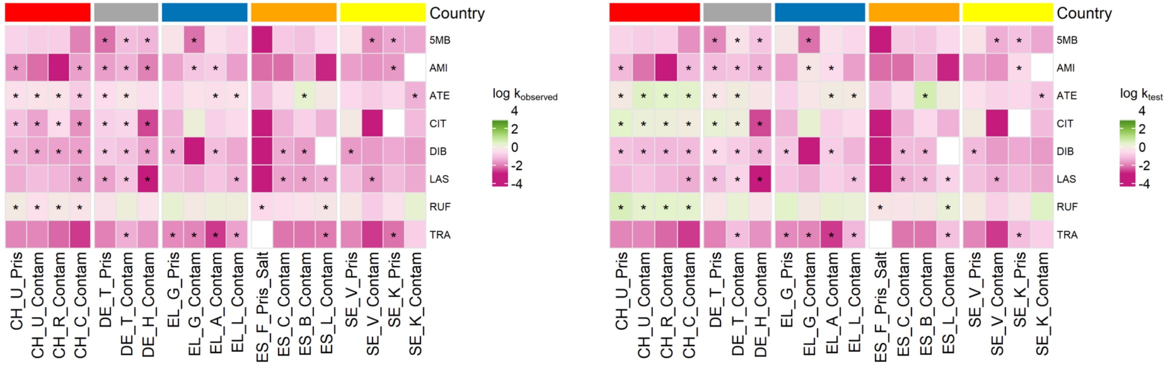

Figure S5. *Clustered heatmaps showing log k (d^{-1}) of compounds that had dissipation in the SC that strongly influenced the estimation of k (marked by *) within at least one given country. A: sorption corrected rate constant ($k_{observed}$), and B: dissipation rate constant derived from the test treatments (k_{test}). Blank cells indicate that k was not available. For elaboration of the country and river segment name abbreviations see Table S1. For elaboration of the compound name abbreviations see Table S3. The results showed sorption correction reduced the spatial variation in rate constants.*

S7. Environmental Parameters

Overall, the biodegradation test mirrored the environmental conditions well and was robust between the three TEST replicates (Figure S6). The conductivity in all incubations was comparable to that in the field (difference < 10%), it was reproducible between three replicates (RSD < 1%), and it was stable during the whole incubation period (RSD < 4%). The pH in all incubations also mirrored well that in the field (difference < 0.3), except for Tegeler Fliess in Germany (pH increased 0.9 before incubation). The Tegeler Fliess had a pH value of 6.8, which was much lower than that for the other rivers (difference > 0.5). Its pH increased rapidly while setting up the experiment (transferring from the sampling bottle to the incubation vessels and mixing with sediment). The increase was most likely due to the outgassing of oversaturated CO_2. The pH remained stable right from the beginning of the incubation in all experiments (standard deviation < 0.2) and reproducible between the three replicates (standard deviation < 0.1). Rapid oxygenation of the water column resulted in an increase in DO to saturation levels (90% to 100%) within the first two hours of incubation. After that, the DO remained constant over the incubation period (RSD < 3%) and reproducible between replicates (RSD < 4%).

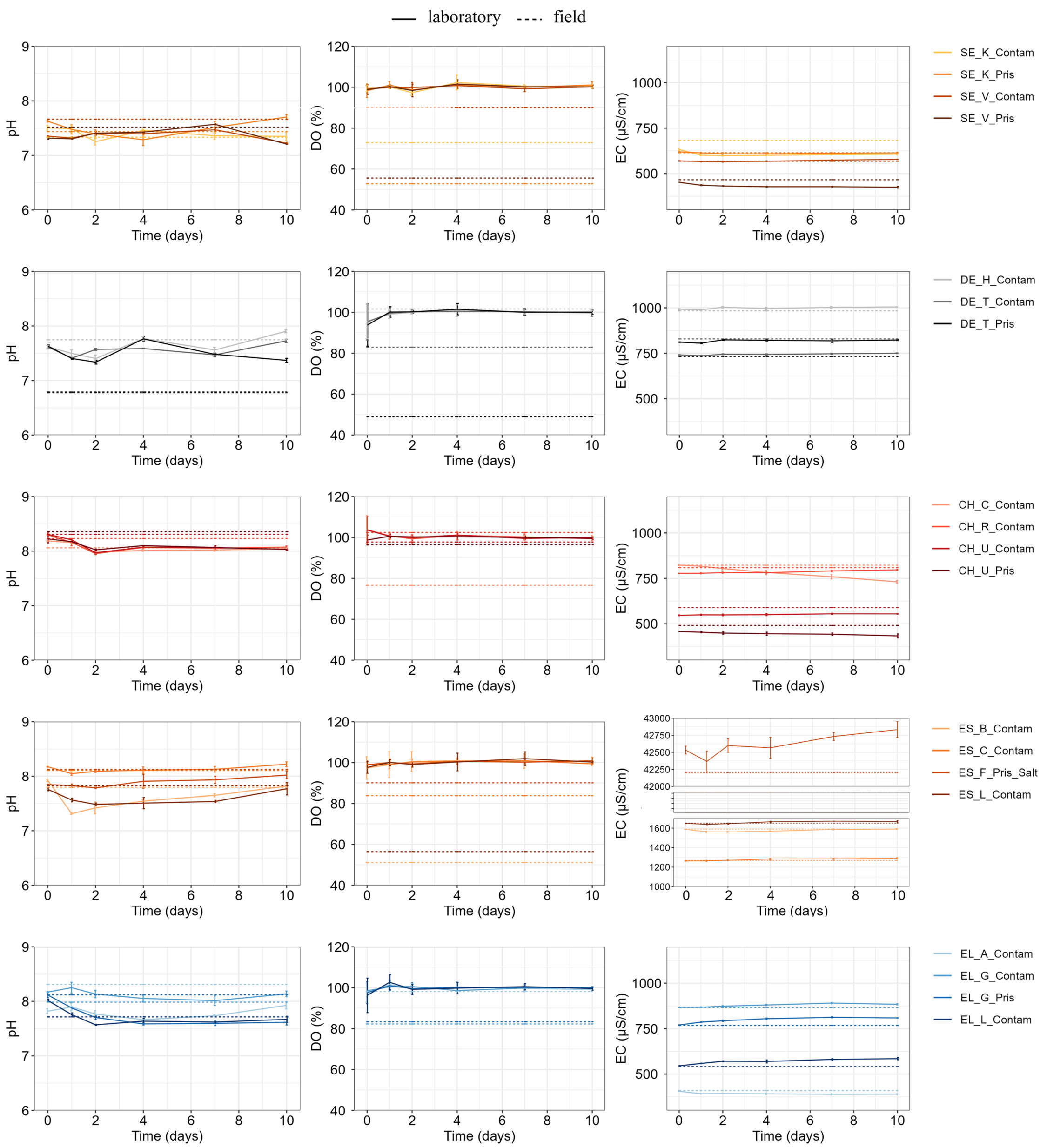

Figure S6. *Trends of pH, dissolved oxygen (DO, mg L^{-1}), and EC (µS cm^{-1}) during each experiment. The average measured values in the field are shown as dashed lines. Each data point is the average of measurements in three test incubations. Error bars represent standard deviations.*

S8. Statistical Results

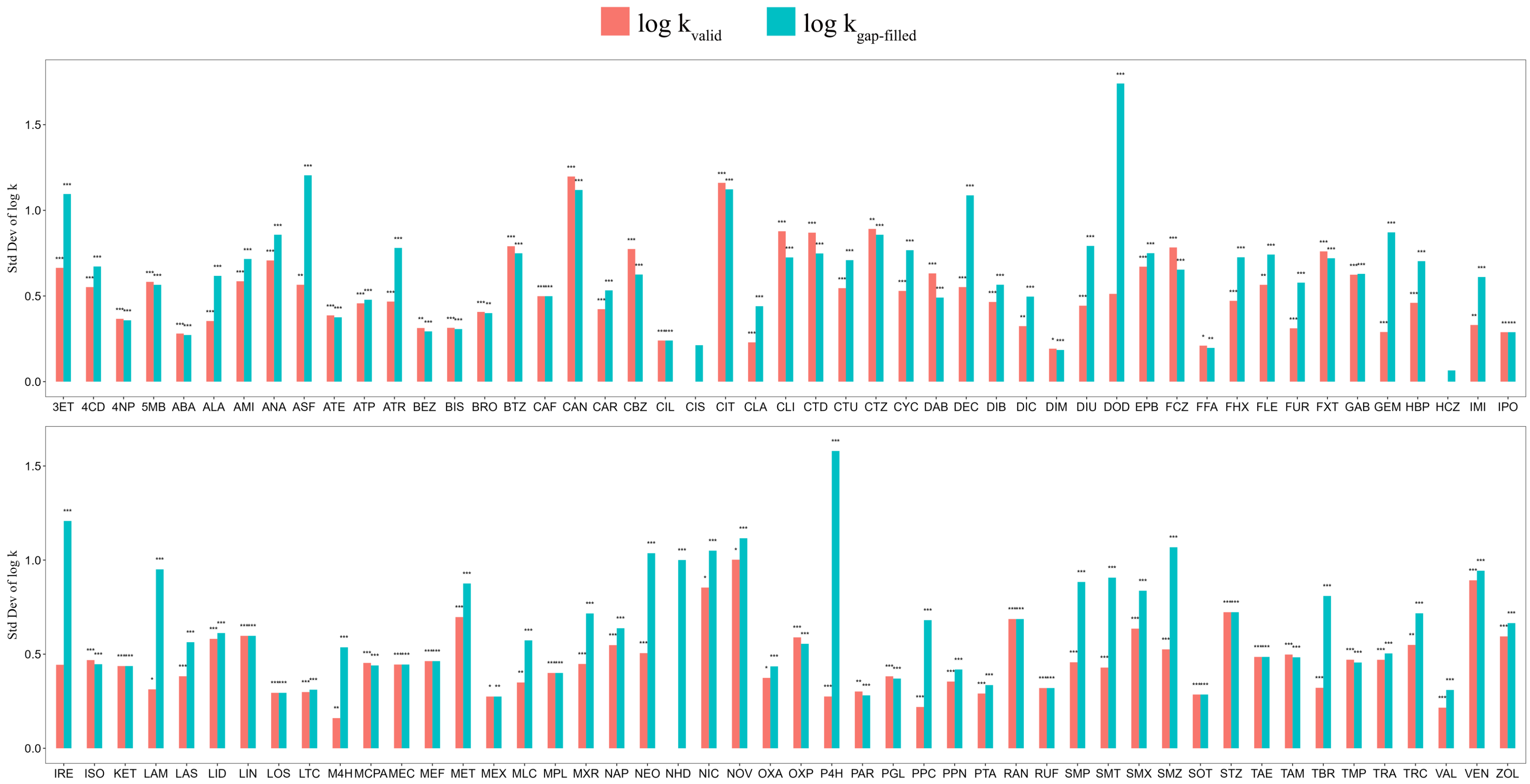

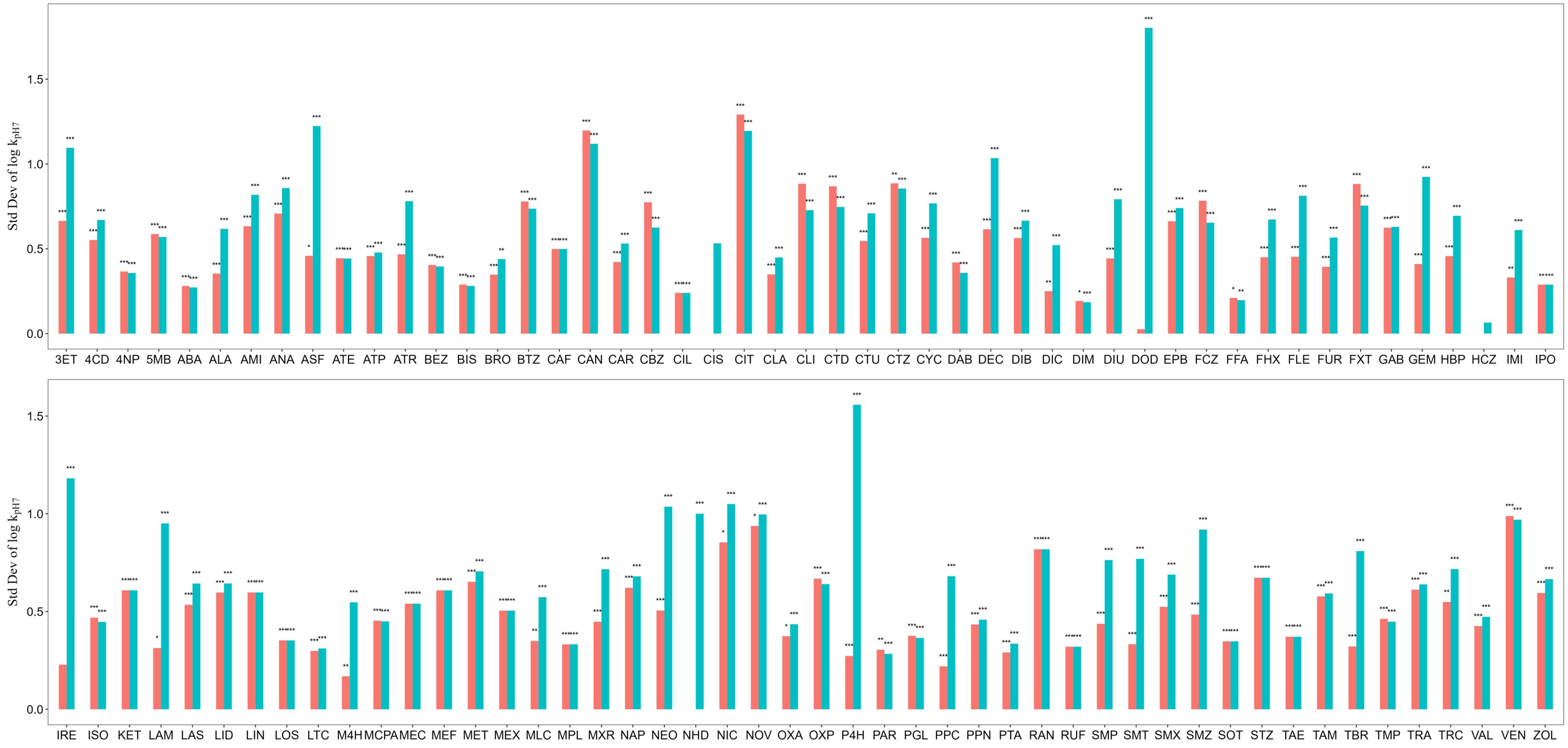

Figure S7. *Histograms displaying the standard deviation (Std Dev) of log k (d^{-1}) and log k_{pH7} (d^{-1}) and the significance of the spatial variation (ANOVA) across all 18 river segments. ANOVA was performed for log k and log k_{pH7} values derived independently for each of the three replicate incubations conducted for each test. Significance is indicated by $P < 0.05$ (*), $P < 0.01$ (**), $P < 0.001$ (***). The color of the column represents the dataset used for the calculations (red: the dataset including only valid k; turquoise: the dataset including gap-filled k).*

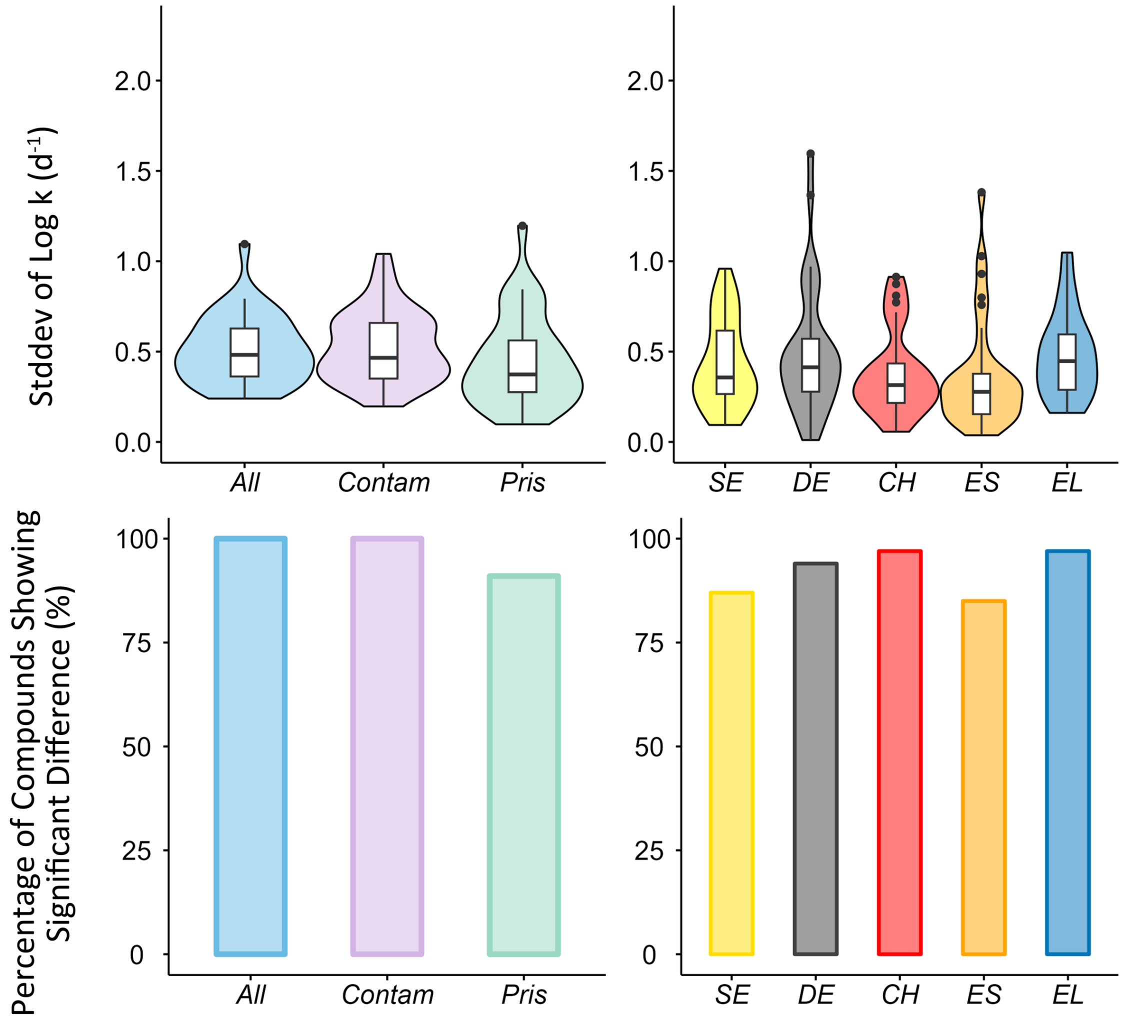

Figure S8. *Violin plots show the median, 25th, and 75th percentiles of the standard deviation (stddev) of log k (d^{-1}). Histograms show the percentage of compounds with significant differences (ANOVA, P < 0.05) across river segments. Results were derived from 18 river segments (All), 13 contaminated river segments (Contam), and five pristine river segments (Pris) across five countries (SE, DE, CH, ES, EL) in Europe, and within each country, respectively. Only compounds that had valid k in at least 12 out of 18 river segments and had k in all 18 river segments after gap-filling were used.*

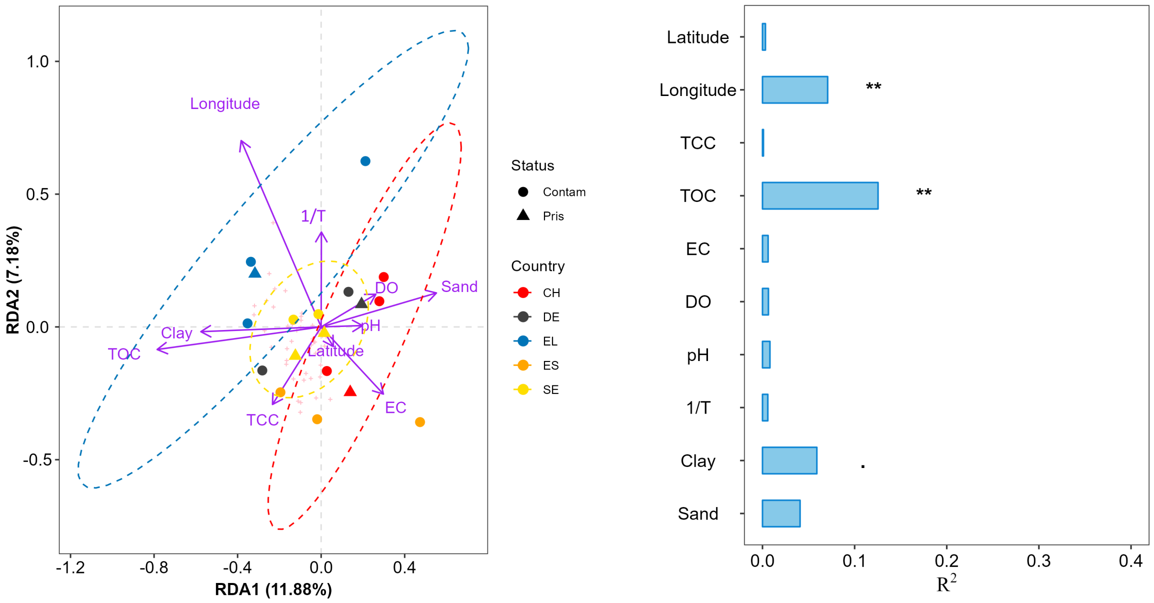

Figure S9. *Redundancy analysis (RDA) shows the relationship between log k (d^{-1}) and 10 environmental factors in 18 river segments in five European countries (SE, DE, CH, ES, EL). The scatterplot shows the first (RDA1) vs. the second (RDA2) dimension. The bar chart presents the percentage of the variance explained (R^2, unbiased, Envfit) for each environmental factor. Only compounds that had valid k in at least 12 river segments were used for the RDA. The significance of the variance explained was represented by: "**" → $P < 0.01$, "*" → $0.01 \leq P < 0.05$, and "." → $0.05 \leq P < 0.1$ (Envfit). The values of all environmental factors except pH and temperature were log-transformed. Temperature was transformed to 1/T (K) to create a distance scale consistent with the Arrhenius relationship.*

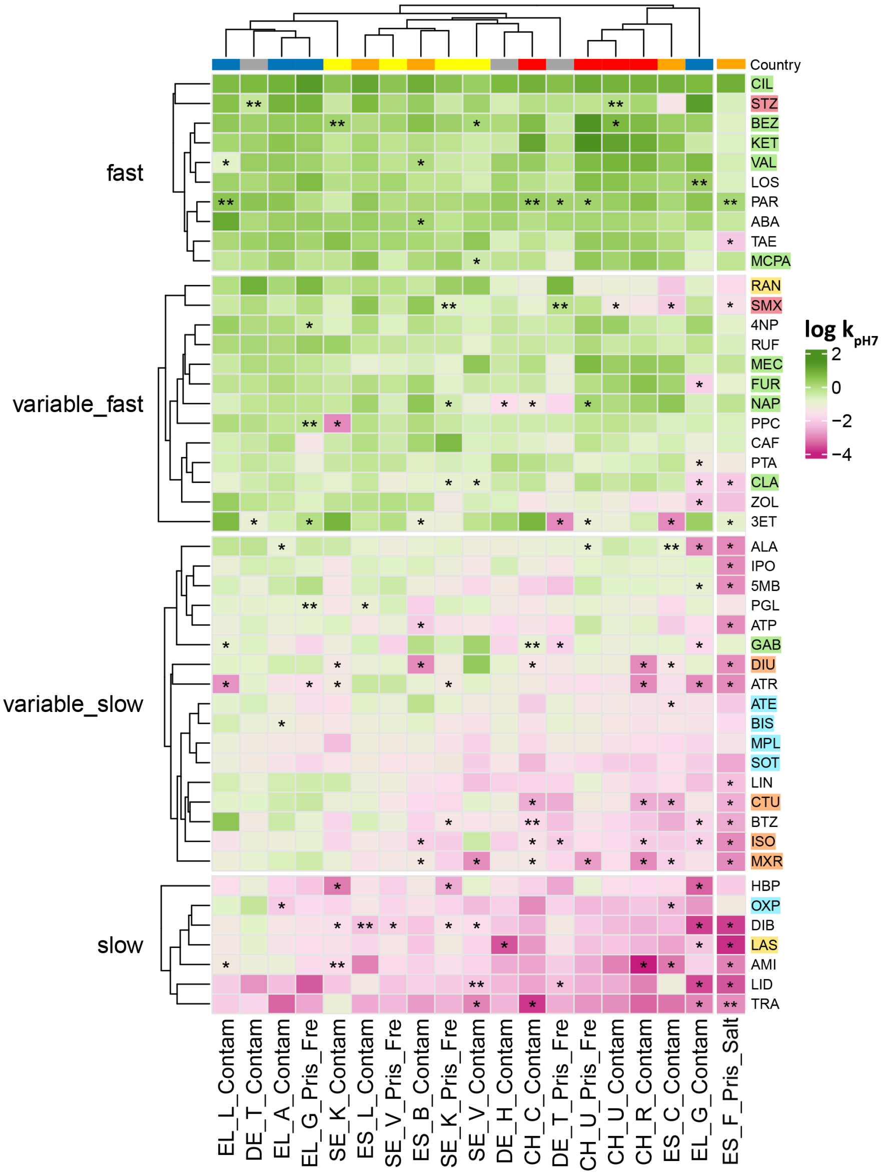

Figure S10. *Clustered heatmap showing log k_{pH7} (d^{-1}) for 47 compounds in 19 river segments (including 13 Contam: contaminated river segments, five Pris: pristine freshwater river segments, and one Pris_Salt: one pristine salty river segment) in five European countries (SE, DE, CH, ES, EL). Invalid k_{pH7} was gap-filled and marked by *, and unavailable k_{pH7} was gap-filled and marked by **. For elaboration of the country and river segment name abbreviations see Table S1. For elaboration of the compound name abbreviations see Table S3.*

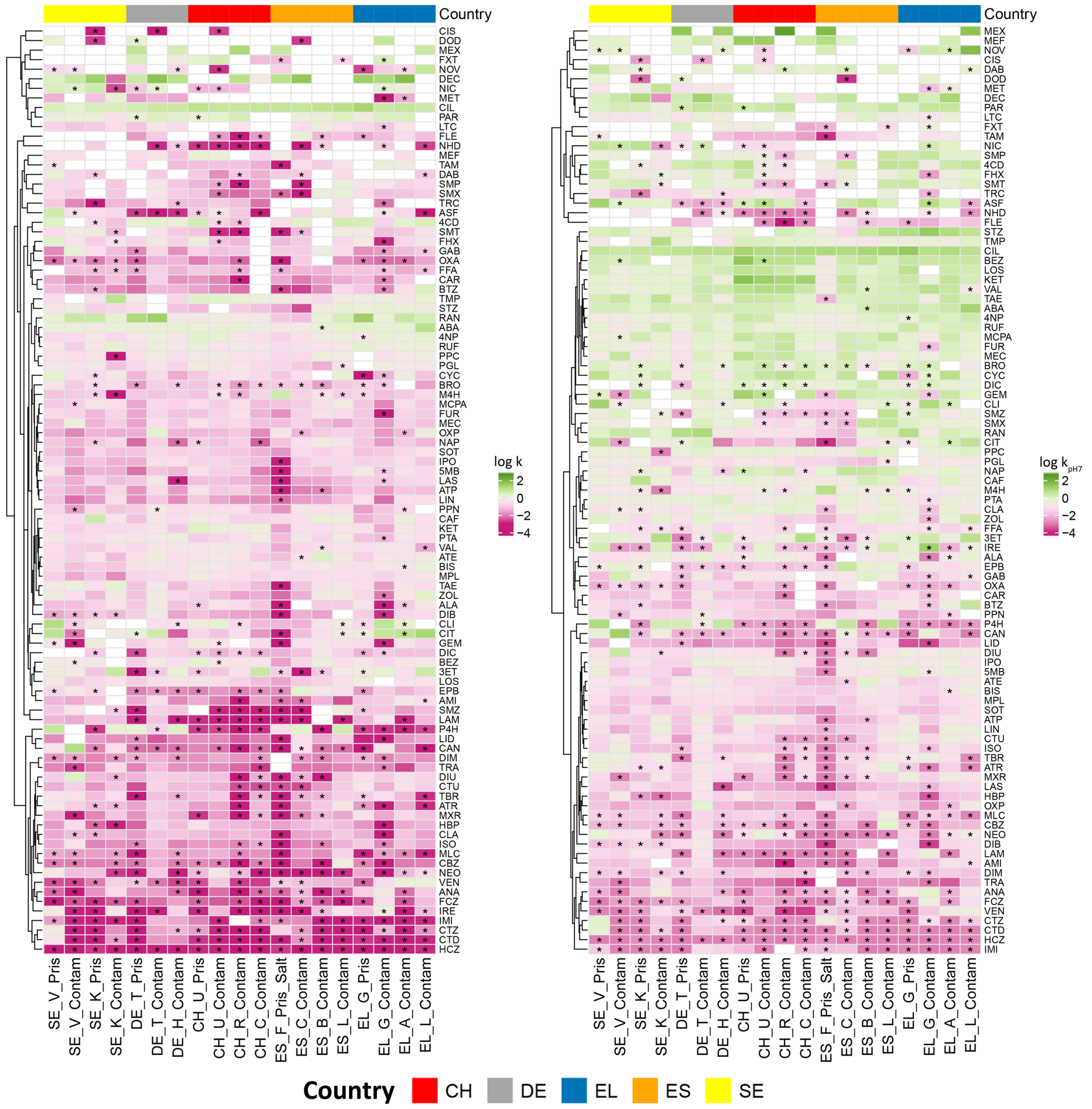

Figure S11. *Clustered heatmaps showing log k (d^{-1}) and log k_{pH7} (d^{-1}) of all compounds in 19 European river segments. Invalid values were gap-filled and marked by *. Blank cells indicate that k was not available. For elaboration of the country and river segment name abbreviations see Table S1. For elaboration of the compound name abbreviations see Table S3.*

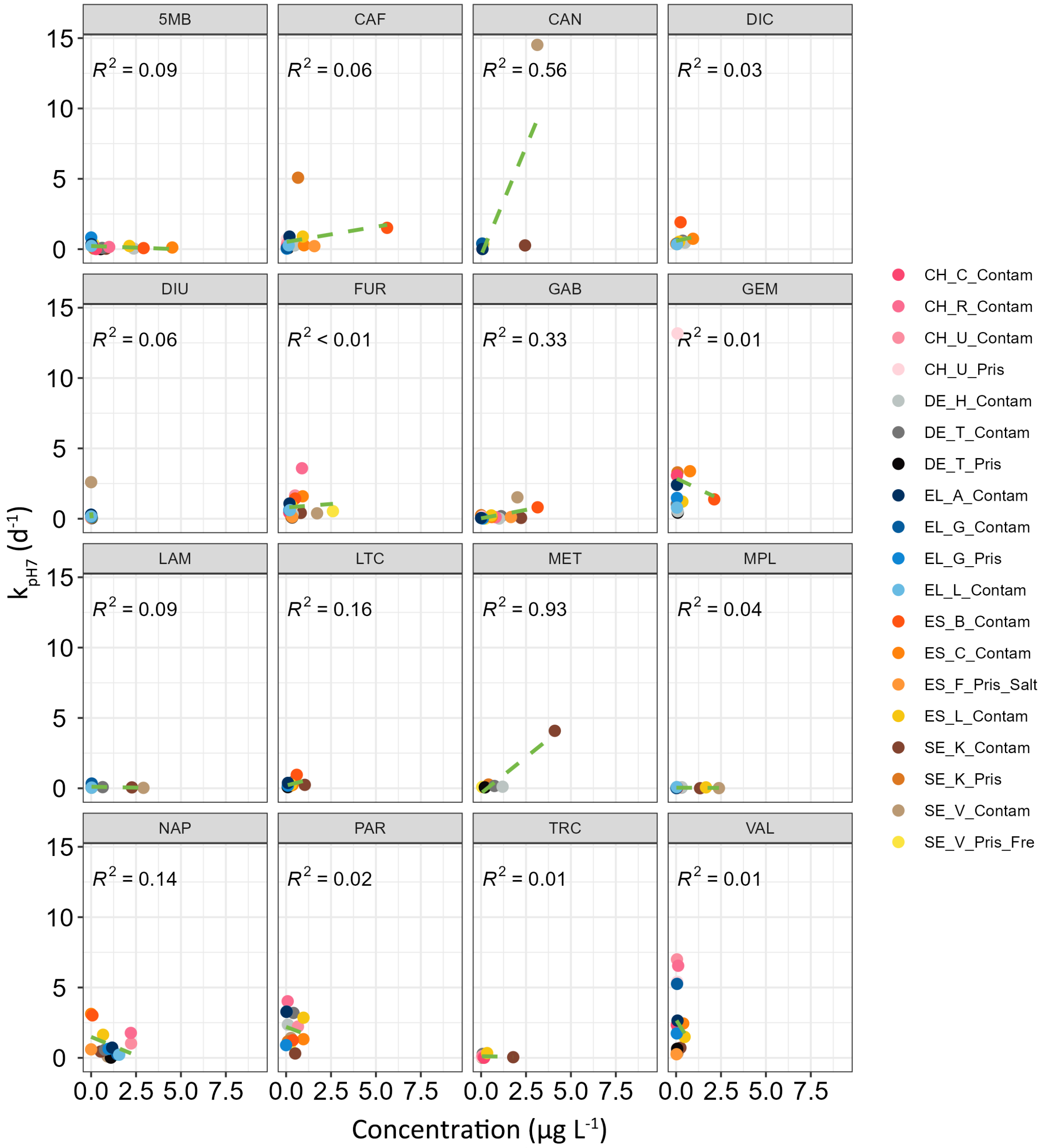

Figure S12. *Correlation between valid k_{pH7} (d^{-1}) and concentration of the compound in the river. All the compounds with a concentration > 1 µg L^{-1} in at least one river segment were tested for the correlation. For elaboration of the country and river segment name abbreviations see Table S1. For elaboration of the compound name abbreviations see Table S3.*

REFERENCES

1. Tian, R.; Posselt, M.; Fenner, K.; McLachlan, M. S. Increasing the Environmental Relevance of Biodegradation Testing by Focusing on Initial Biodegradation Kinetics and Employing Low-Level Spiking. *Environ. Sci. Technol. Lett.* **2023**, 10 (1), 40–45. https://doi.org/10.1021/acs.estlett.2c00811.
2. Tian, R.; Posselt, M.; Miaz, L. T.; Fenner, K.; McLachlan, M. S. Influence of Season on Biodegradation Rates in Rivers. *Environ. Sci. Technol.* **2024**, 58 (16), 7144–7153. https://doi.org/10.1021/acs.est.3c10541.
